# Modelling human adult V-SVZ niche assembly and ependymal cell generation in brain organoids

**DOI:** 10.1038/s44319-025-00621-3

**Published:** 2025-11-05

**Authors:** Styliani Vassalou, Maria-Eleni Lalioti, Rossella Di Giaimo, Stavros Taraviras, Silvia Cappello, Christina Kyrousi

**Affiliations:** 1 First Department of Psychiatry, Medical School, National and Kapodistrian University of Athens, Athens, 11527 Greece; 2 University Mental Health, Neurosciences and Precision Medicine Research Institute “Costas Stefanis”, Athens, 11527 Greece; 3 Department of Physiology, School of Medicine, University of Patras, Patras, 26504 Greece; 4 Max Planck Institute of Psychiatry, Munich, 80804 Germany; 5 Department of Biology, University of Naples Federico II, Naples, 80126 Italy; 6 Division of Physiological Genomics, Biomedical Center (BMC), Faculty of Medicine, Ludwig Maximilian University (LMU), Munich, 82152 Germany

**Keywords:** Brain Organoids, GEMC1-MCIDAS Pathway, Human Multiciliated Ependymal Cells, Human V-SVZ Niche Assembly, Hypocellular Gap, Development, Neuroscience, Stem Cells & Regenerative Medicine

## Abstract

The V-SVZ niche is vital for adult neurogenesis in mammals, yet its regulation in humans remains poorly understood. Current models, including brain organoids, fail to replicate the unique cytoarchitecture of this niche, particularly the multiciliated ependymal cells, which are essential for its function and organization. Here, we utilize GEMC1 and MCIDAS to program human apical radial glial cells into ependymal cells, employing human brain organoids as a model. This approach induces premature ependymal cell differentiation and reorganization of the embryonic neurogenic niche, conferring characteristics of the human adult V-SVZ niche. Our findings highlight a molecular pathway that leads to ependymal cell generation and adult human V-SVZ niche reconstruction, providing a platform to study its development and function.

## Synopsis

Human progenitors are programmed into ependymal cells in brain organoids via GEMC1 and MCIDAS expression. This strategy recapitulates hallmarks of the adult human V-SVZ niche, providing a platform to investigate its regulation and function.

- GEMC1 and MCIDAS induce human ependymal cell generation.
- Programmed ependymal cells exhibit intrinsic human-specific characteristics.
- Ependymal cell generation promotes human adult V-SVZ niche assembly.

## Abstract (web summary)

Human progenitors are programmed into ependymal cells in brain organoids via GEMC1 and MCIDAS expression. This strategy recapitulates hallmarks of the adult human V-SVZ niche, providing a platform to investigate its regulation and function.

## Graphical abstract

**Figure:**
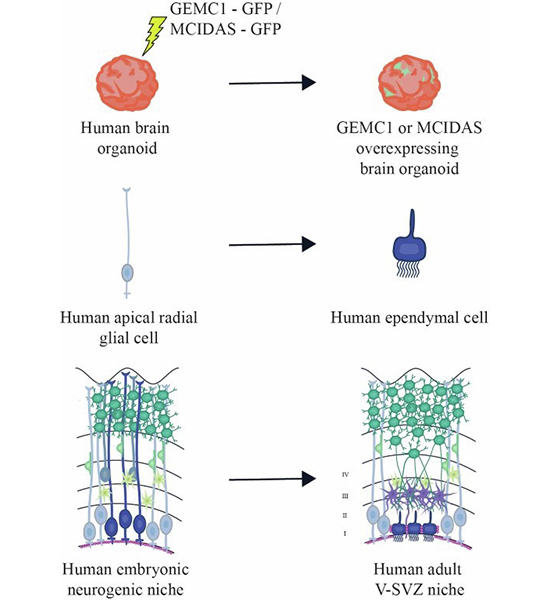

**Figure:**
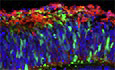

## Introduction

The adult ventricular-subventricular zone (aV-SVZ) of the lateral ventricles is one of the main neurogenic regions in the adult mammalian brain where new neurons are produced throughout life (Gage, 2000), especially in species reliant on processing olfactory information (Alvarez-Buylla and García-Verdugo, 2002). In humans, active adult neurogenesis remains controversial, while the niche formation has not been extensively studied. The human aV-SVZ (haV-SVZ) niche is divided into four layers: the ependymal layer (Layer I), the hypocellular gap (Layer II), the astrocytic ribbon (Layer III), and the transitional layer (Layer IV) (Altmann et al, 2019; Ioannidis et al, 2021). Layer I is a monolayer of ependymal cells (hECs) that have multiple cilia protruding into the lumen and extend a basal process perpendicularly to Layer II. This short basal process distinguishes hECs from those of mice (Doetsch et al, 1999). Layer II is a region devoid of cell somata, composed of the cytoplasmic expansions of ECs, aNSCs, and neurons, and its formation starts 6 months after birth (Akter et al, 2020; Quiñones-Hinojosa et al, 2006; Sawamoto et al, 2011). Layer III contains astrocytic cells, like aNSCs, which are organized in a ribbon (Sanai et al, 2004) with self-renewal and differentiation abilities (Johansson et al, 1999; Kukekov et al, 1999), while some of them extend a process through Layer I to the lateral ventricles (Sanai et al, 2004). Lastly, Layer IV is composed of myelinated axons and oligodendrocytes (Altmann et al, 2019).

During aV-SVZ assembly, aNSCs and ECs play a crucial role. Ιn mice, both cell types derive from radial glial cells (RGs), which become fate-restricted during mid-embryogenesis undergoing a transformation process at late embryonic stages and promote the niche reconstruction (Fuentealba et al, 2015; Merkle et al, 2004; Spassky et al, 2005). At early postnatal stages, RGs initially generate aNSCs, while upon terminal differentiation they generate ECs (Ortiz-Álvarez et al, 2019).

GEMC1 and MCIDAS have been identified as crucial regulators of EC fate acquisition. In mice, GEMC1 acts upstream of MCIDAS and is responsible for the commitment of RGs to EC fate, whilst MCIDAS upon its activation from GEMC1 is necessary for the completion of the EC differentiation (Kyrousi et al, 2015; Ma et al, 2014; Terré et al, 2016). Both proteins interact with E2F4/5 to induce the expression of C-MYB and CCNO, which are necessary for centriole amplification (Arbi et al, 2017; Funk et al, 2015; Tan et al, 2013), while they also induce the expression of the transcription factors P73 and FOXJ1, which are crucial for the formation and cell membrane docking of ECs’ basal bodies (Jacquet et al, 2009; Kyrousi et al, 2015; Lalioti et al, 2019b; Marshall et al, 2016). In addition, the GEMC1/MCIDAS pathway is activated during the differentiation of other multiciliated cells, the choroid plexus, and non-multiciliated cells, the Cajal-Retzius neurons (Lewis and Stracker, 2021; Moreau et al, 2023).

The cell-non-autonomous role of the EC differentiation in the normal formation of the aV-SVZ niche has already been shown in the murine model. ECs have been accounted for the ventricular growth that occurs during late embryonic brain development and the proper aV-SVZ niche assembly, through the influence of cell–cell adhesions, and their depletion results in disorganization of the niche and impaired adult neurogenesis (Quaresima et al, 2022; Redmond et al, 2019; Paez-Gonzalez et al, 2011; Wang et al, 2014). *GEMC1* and *MCIDAS* mutations have been identified in patients with congenital hydrocephalus, a phenotype that is recapitulated in mice (Lalioti et al, 2019a; Lu et al, 2019; Robson et al, 2020). In humans, a recent study indicates that ependymogenesis is initiated at 21 gestational week and is completed ~10 days after birth (Coletti et al, 2018), however, the cellular and molecular mechanisms regulating haV-SVZ niche assembly have not yet been elucidated.

Toward deciphering haV-SVZ niche assembly, we sought to investigate how the molecular mechanisms that regulate ECs’ generation could influence the formation of the niche. Employing human brain organoids (hBOs), we show that the GEMC1/MCIDAS pathway, upon ectopic activation in human apical RGs (haRGs) leads to ECs’ premature generation. Our data showcase for the first time a model that generates ECs with human-specific morphological characteristics, while ECs’ premature differentiation results in changes in the cytoarchitecture of the embryonic niche, which adapts aV-SVZ niche characteristics.

## Results and discussion

### Ectopic expression of *GEMC1* and *MCIDAS* in human aRGs influences their fate decisions

In recent years, hBOs have been used as an innovative tool to study brain development, evolution, and disease, resembling their organization, cellular composition, and gene expression. Their ability to model lineage progression, differentiation, and maturation makes them invaluable for studying neurodevelopmental and neurodegenerative disorders (Damianidou et al, 2022; Kyrousi and Cappello, 2020). Despite extensive research, the specification and formation of haV-SVZ remains unclear.

ECs are essential for aV-SVZ cytoarchitecture in mice thus, we sought to investigate whether hECs are also important components in haV-SVZ niche assembly. Towards this, we studied the expression and function of *GEMC1* and *MCIDAS*, regulators of EC fate acquisition in mice (Kyrousi et al, 2015, 2016, 2017; Lalioti et al, 2019b, 2019a). Taking advantage of published scRNA-seq data from hBOs (Uzquiano et al, 2022; Data ref: Uzquiano et al, 2022), we investigated *GEMC1* and *MCIDAS* expression during early and late BO development. More specifically, we examined their cell type-specific expression pattern and we show that they exhibit low expression levels in 2- and 6 month  (m) old BOs (Fig. EV1A–B’). We validated these data by qPCR on RNA isolated from BOs at different developmental stages. Similar to the scRNA-seq data, *GEMC1* and *MCIDAS* expression spiked at early development, at 40- and 50 day (d) old organoids, respectively, stages that correspond to the early fetal human brain (8–10 post-conception weeks) but rapidly declined at low basal expression levels. Interestingly, both genes show increasing expression at 7 m BOs, a stage which possibly corresponds to a mid-late fetal developmental stage in vivo (Fig. EV1C,D). These organoid-derived data could indicate that *GEMC1* and *MCIDAS* are not highly expressed in the human brain during early development, but may show more robust expression during later developmental stages, and that their expression is delayed compared to mice.

**Figure EV1:**

Cell- and time-specific expression patterns of GEMC1 and MCIDAS and their influence on aRGs number. (**A**–**B’**) Analyses of published single-cell RNA sequencing data in cells isolated from hBOs in different developmental stages (Data ref: Uzquiano et al, 2022). Violin plots showing the GEMC1 and MCIDAS normalized counts at different cell types in 2 m (**A**, **B**) and 6 m hBOs (**A’**, **B’**). (**C**, **D**) GEMC1 and MCIDAS quantitative mRNA expression analyses in hBOs in different developmental stages. Data normalized against GADPH. Three independent replicates were analyzed, each bearing the homogenate of three individual brain organoids. (**E**–**J”**) GFP, *GEMC1* or *MCIDAS* OE BOs at 4 dpe stained using antibodies against GFP and Ki-67 (**E**–**G”**) or PAX6 (**H**–**J**”). Data are represented as the mean ± s.e.m. Statistical analysis was performed using the unpaired *t* test (*P < 0.05, ***P < 0.001, ****P < 0.0001). White boxes indicate the area zoomed-in in the corresponding pictures. Arrows indicate Ki-67+GFP+ or PAX6+GFP+ cells. Scale bars: 30 μm.

**Figure 1:**
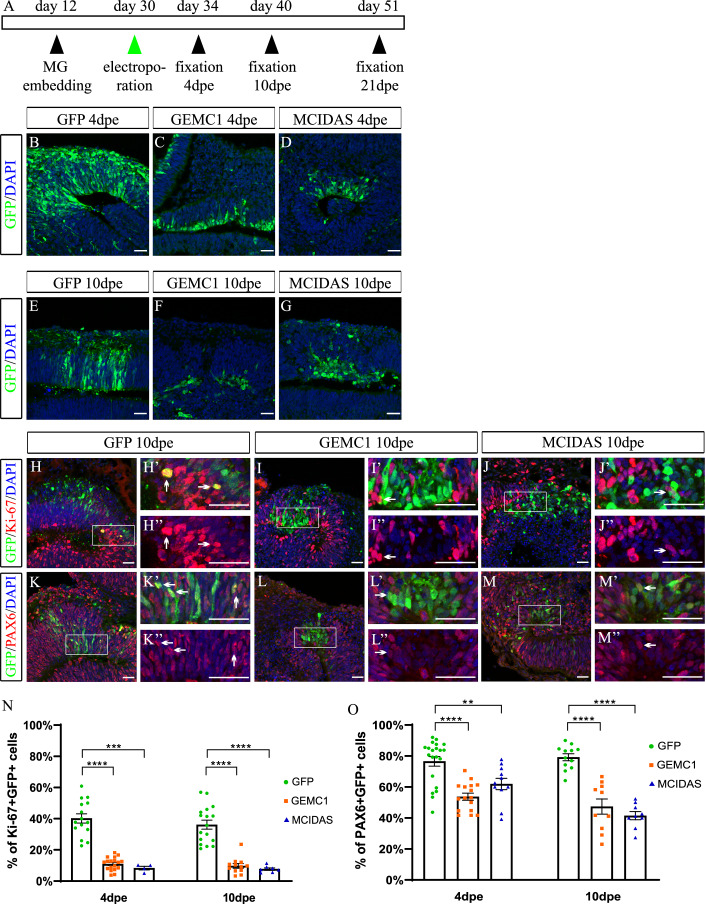
Reduced numbers of aRGs upon *GEMC1* and *MCIDAS* OE in hBOs. (**A**) Schematic of the experimental procedure. (**B**–**G**) GFP, *GEMC1* or *MCIDAS* OE BOs at 4- (**B**–**D**) and 10- (**E**–**G**) dpe stained using an antibody against GFP. Biological replicates 4 dpe: GFP: v = 21, GEMC1: v = 18, MCIDAS: v = 11, 10 dpe: GFP: v = 23, GEMC1: v = 26, MCIDAS: v = 14. (**H**–**M”**) GFP, *GEMC1* or *MCIDAS* OE BOs at 10 dpe stained using antibodies against GFP and Ki-67 (**H**–**J”**) or PAX6 (**K**–**M”**). (**N**, **O**) Quantification of the percentage of GFP+ cells that co-express Ki-67 (**N**) or PAX6 (**O**). Biological replicates: Ki-67 4 dpe: GFP: v = 15, GEMC1: v = 18, MCIDAS: v = 4, 10 dpe: GFP: v = 17, GEMC1: v = 12, MCIDAS: v = 7 (*P* value: <0.0001, 0.0005, <0.0001 and <0.0001, respectively). PAX6 4 dpe: GFP: v = 21, GEMC1: v = 16, MCIDAS: v = 11, 10 dpe: GFP: v = 12, GEMC1: v = 10, MCIDAS: v = 9 (*P* value: <0.0001, 0.0062, <0.0001 and <0.0001, respectively). White boxes indicate the area zoomed-in in the corresponding pictures. Arrows indicate Ki-67+GFP+ or PAX6+ GFP+ cells. Data are represented as the mean ±s.e.m. Statistical analysis was performed using the nonparametric two-tailed Mann–Whitney test (**P < 0.01, ***P < 0.001, ****P < 0.0001). Scale bars: 30 μm. MG Matrigel. Source data are available online for this figure.

Given the role of these factors on cell fate commitment in mice, we speculated that they could be influential on haRGs fate acquisition. Toward this, we ectopically overexpressed (OE) *GEMC1* and *MCIDAS* in hBOs in a stage that corresponds to early fetal corticogenesis in humans. BOs were injected in their ventricle-like structures with plasmids expressing *GEMC1* or *MCIDAS* together with GFP (GEMC1 and MCIDAS respectively) or GFP alone (GFP) followed by electroporation to target aRGs at the ventricular zone-like areas (Rapti and Kyrousi 2025). The electroporation was performed on 30 d BOs, a stage in which *GEMC1* and *MCIDAS* endogenous expressions are very low (Fig. EV1C,D) and under normal conditions aRGs typically generate neurons. BOs were analyzed several days (4, 10, 21) post electroporation (dpe) to follow the differentiation potential of aRGs longitudinally (Fig. 1A). *GEMC1* and *MCIDAS* OE cells at 4- and 10 dpe showed an altered shape which did not resemble the typical radial morphology of aRGs (Fig. 1B–G). To further investigate the cellular identity of the OE cells, we used Ki-67 to label the cycling progenitors (Figs. 1H–J” and EV1E–G”) and PAX6 to visualize the aRGs and bRGs (Figs. 1K–M” and EV1H–J”). Both *GEMC1* and *MCIDAS* OE led to decreased numbers of Ki-67+GFP+ cells at 4- and 10 dpe (Fig. 1N), indicating an instant and significant drop in the proliferation rate of the cells. Similarly, *GEMC1* and *MCIDAS* OE resulted in a reduced number of PAX6+GFP+ RGs at 4- and 10 dpe (Fig. 1O). Taken together, our data show that upon *GEMC1* and *MCIDAS* OE haRGs undergo a cell fate switch, losing their characteristic radial morphology and innate proliferation capacity. These results come in line with previous findings in the murine brain (Kyrousi et al, 2015).

**Figure 2:**
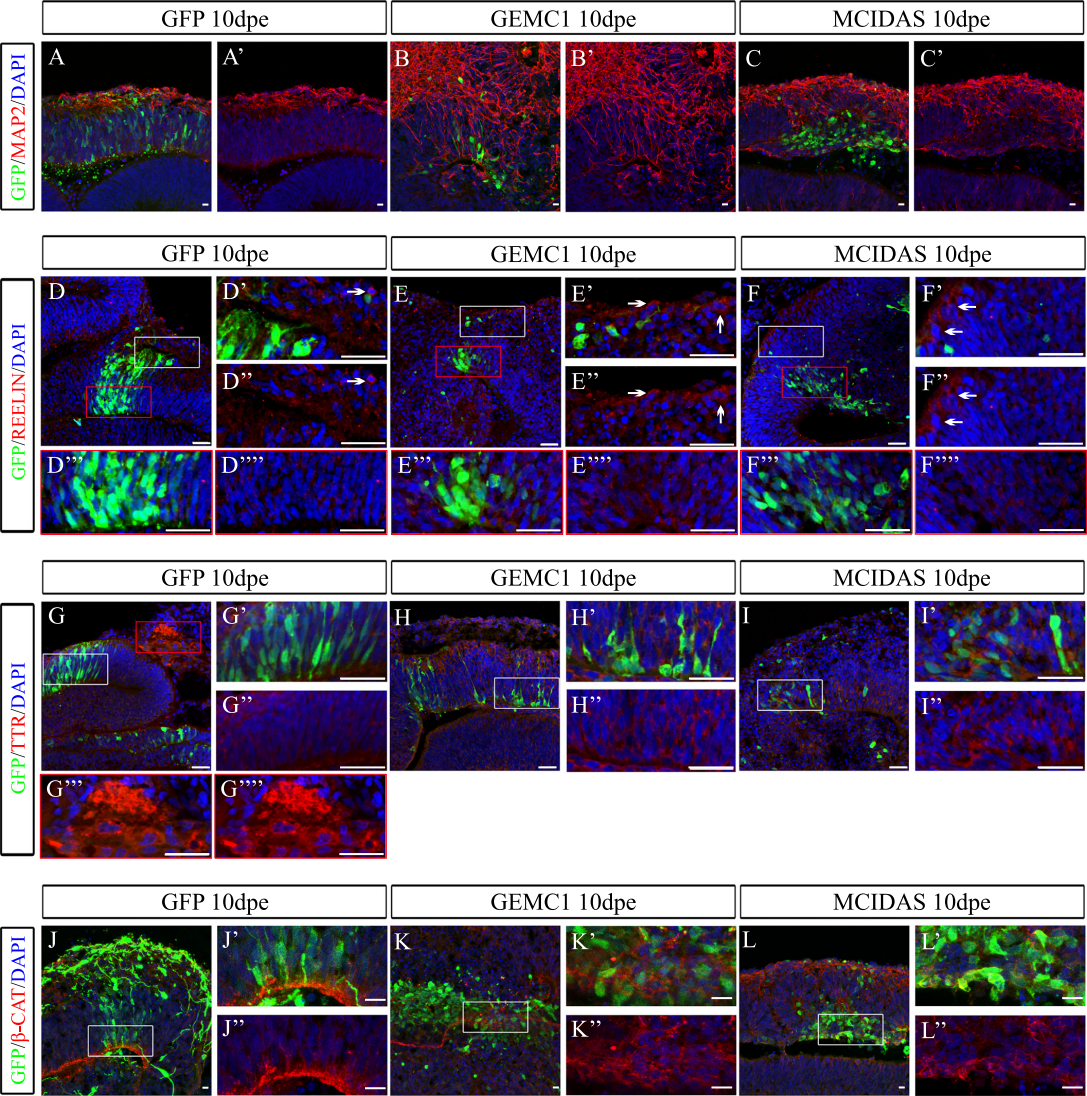
*GEMC1* and *MCIDAS* OE affects the cytoarchitecture of the ventricular zone-like areas. (**A**–**I”**) GFP, *GEMC1* or *MCIDAS* OE BOs at 10 dpe stained using antibodies against GFP and MAP2 (**A**–**C’**), REELIN (**D**–**F””**), TTR (**G**–**I”**) or β-catenin (**J**–**L”**). Biological replicates: MAP2: GFP: v = 2, GEMC1: v = 3, MCIDAS: v = 3. REELIN: GFP: v = 3, GEMC1: v = 3, MCIDAS: v = 3. TTR: GFP: v = 3, GEMC1: v = 2, MCIDAS: v = 3. β-cat: v = 2 for all conditions. White and red boxes indicate the area zoomed-in in the corresponding pictures. Scale bars: 30 μm. Source data are available online for this figure.

Under physiological conditions, haRGs differentiate into neurons; however, our results indicate that *GEMC1* and *MCIDAS* OE cells experienced cell fate changes. Thus, we sought to investigate whether GEMC1 and MCIDAS affect the neuronal output in BOs. To this end, we stained the *GEMC1* and *MCIDAS* OE BOs with the neuronal markers MAP2 and NEUN and show that the OE cells were not MAP2+ or NEUN+ , indicating that they do not acquire a neuronal identity (Figs. 2A–C’ and EV2A–F”). Nevertheless, we noticed numerous MAP2+ neuronal processes intruding into the apical areas of the embryonic neuroepithelium at 4- (Fig. EV2A–C’) and 10 dpe (Fig. 2A–C’), indicating a substantial modification of the niche’s cytoarchitecture, while the neuronal somata (NEUN+ cell bodies) remain restricted into the cortical plate (Fig. EV2D–F”). Given that the GEMC1/MCIDAS pathway is activated during Cajal-Retzius neurons and choroid plexus cells differentiation (Lewis and Stracker, 2021; Moreau et al, 2023), we investigated whether the OE cells expressed their cell markers. We did not observe REELIN+GFP+ (Fig. 2D–F””) nor TTR+GFP+ (Fig. 2G–I”) cells in the *GEMC1* and *MCIDAS* OE BOs, indicating that the OE aRGs did not acquire Cajal-Retzius nor choroid plexus cell identities, respectively. Lastly, aiming for an overall characterization of the manipulated cells, we assessed the expression of other brain cell markers, namely OLIG2, a marker of oligodendrocytes. However, we did not observe OLIG2+GFP+ cells in the *GEMC1* and *MCIDAS* OE BOs (Fig. EV2G–I”).

**Figure EV2:**

GEMC1 and MCIDAS OE cells fail to differentiate into neurons. (**A**–**C’**) GFP, *GEMC1* or *MCIDAS* OE BOs at 4 dpe stained using antibodies against GFP and MAP2. (**D**–**F”**) GFP, *GEMC1* or *MCIDAS* OE BOs at 10 dpe stained using antibodies against GFP and NEUN. (**G**–**I”**) GFP, *GEMC1* or *MCIDAS* OE BOs at 21 dpe stained using antibodies against GFP and OLIG2. Biological replicates: MAP2: GFP: v = 9, GEMC1: v = 4, MCIDAS: v = 4. NEUN: v = 3 for all conditions. OLIG2: GFP: v = 1, GEMCI: v = 2, MCIDAS: v = 2. White and red boxes indicate the area zoomed-in in the corresponding pictures. Arrows indicate NEUN+GFP+ or OLIG2+ cells. Scale bars: 30 μm.

To further investigate the cellular structure of the OE V-SVZ niche, we examined whether the integrity of the apical belt is being compromised. Indeed, we noticed that upon *GEMC1* and *MCIDAS* OE the distribution of β-catenin (β-cat) is changed (Fig. 2J–L”), suggesting a redistribution of the adhesion proteins, indicating a niche cytoarchitecture remodeling. Overall, our data show that GEMC1 and MCIDAS OE reconfigured the embryonic neurogenic region and altered neural progenitor lineage in hBOs.

### *GEMC1* and *MCIDAS* OE induce haRGs differentiation into the EC lineage

**Figure 3:**
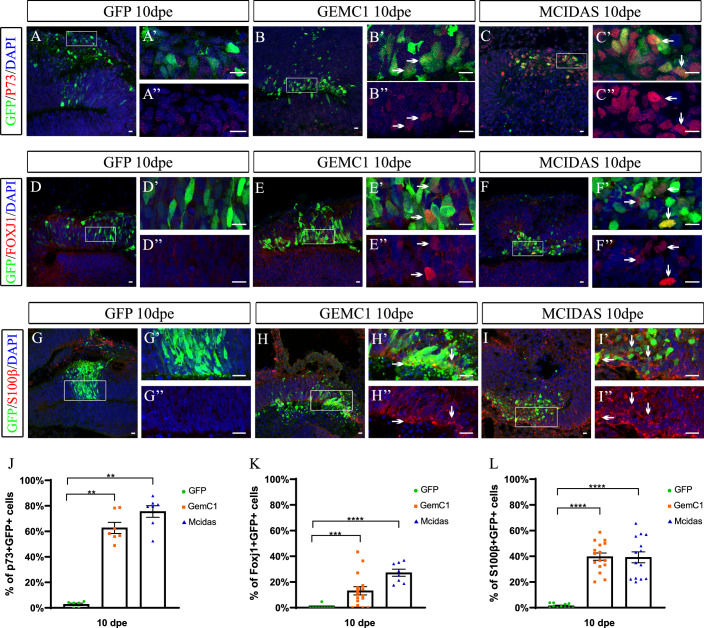
*GEMC1* and *MCIDAS* OE results in hECs differentiation. (**A**–**I”**) GFP, *GEMC1* or *MCIDAS* OE BOs at 10 dpe were stained using antibodies against GFP and P73 (**A**–**C”**), FOXJ1 (**D**–**F”**) or S100β (**G**–**I”**). (**J**–**L**) Quantification of the percentage of P73+GFP+ (**J**), FOXJ1+GFP+ (**K**) and S100β+GFP+ cells (**L**). Biological replicates: P73: GFP: v = 6, GEMC1: v = 7, MCIDAS: v = 7 (*P* value: 0.0012 and 0.0012, respectively). FOXJ1: GFP: v = 10, GEMC1: v = 16, MCIDAS: v = 8 (*P* value: 0.0003 and <0.0001, respectively). S100β: GFP: v = 11, GEMC1: v = 16, MCIDAS: v = 14 (*P* value: <0.0001 and <0.0001, respectively). Data are represented as the mean ± s.e.m. Statistical analysis was performed using the nonparametric two-tailed Mann–Whitney test (**P < 0.01, ***P < 0.001, ****P < 0.0001). White boxes indicate the area zoomed-in in the corresponding pictures. Arrows indicate P73+GFP+ cells, FOXJ1+GFP+ cells or S100β+GFP+ cells. Scale bars: 10 μm. Source data are available online for this figure.

**Figure EV3:**
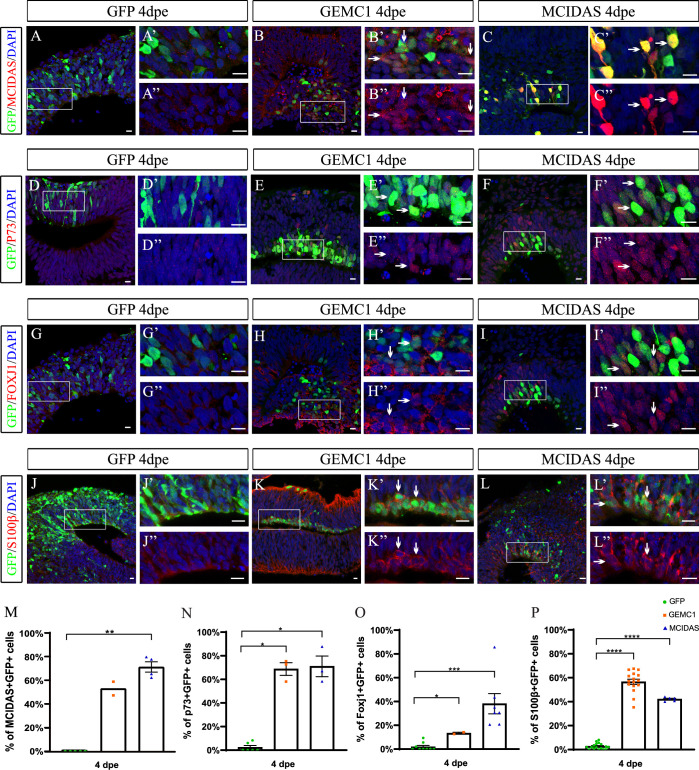
GEMC1 and MCIDAS OE cells express ECs markers. (**A**–**L”**) GFP, *GEMC1* or *MCIDAS* OE BOs at 4 dpe stained using antibodies against GFP and MCIDAS (**A**–**C”**), P73 (**D**–**F”**), FOXJ1 (**G**–**I”**) or S100β (**J**–**L”**). DAPI and GFP channels in images (**A**–**A”**) are reused in (**G**–**G”**), DAPI and GFP channels in images (**B**–**B”**) are reused in (**H**–**H”**) and DAPI and GFP channels in images (**F**–**F”**) are reused in (**I**–**I”**). (**M**–**P**) Quantification of the percentage of MCIDAS+GFP+ (**M**), P73+GFP+ (**N**), FOXJ1+GFP+ (**O**) and S100β+GFP+ cells (**P**). Biological replicates: MCIDAS: GFP: v = 5, GEMC1: v = 2, MCIDAS: v = 4 (*P* value: 0.0079). P73: GFP: v = 6, GEMC1: v = 3, MCIDAS: v = 3 (*P* value: 0.0119 and 0.0119, respectively). FOXJ1: GFP: v = 9, GEMC1: v = 2, MCIDAS: v = 7 (*P* value: 0.0182 and 0.0002, respectively). S100β: GFP: v = 17, GEMC1: v = 16, MCIDAS: v = 5 (*P* value: <0.0001 and <0.0001, respectively). Data are represented as the mean ± s.e.m. Statistical analysis was performed using the nonparametric two-tailed Mann–Whitney test (*<*P* < 0.05, **P < 0.01, ***P < 0.001, ****P < 0.0001). White boxes indicate the area zoomed-in in the corresponding pictures. Arrows indicate MCIDAS+GFP+ , P73+GFP+ cells, FOXJ1+GFP+ cells or S100β+GFP+ cells. Scale bars: 10 μm.

In mice, GEMC1 and MCIDAS promote RGs’ commitment and differentiation to ECs by activating the molecular pathway responsible for multiciliated cells differentiation (Kyrousi et al, 2015; Arbi et al, 2016; Lalioti et al, 2019b, 2019a). Nevertheless, the molecular pathway leading to hECs generation has not been described. To delve into this, we OE *GEMC1* and *MCIDAS* in hBOs and examined whether key players of the multiciliation program are activated. In mice, it was shown that GEMC1 acts upstream of MCIDAS (Arbi et al, 2016; Kyrousi et al, 2015; Terré et al, 2016). In line with this, we observed ectopically elevated levels of MCIDAS in *GEMC1* OE BOs at 4 dpe (Fig. EV3A-C”, M). We also checked other downstream molecules responsible for multiple cilia generation in cells, namely P73 and FOXJ1 (Figs. 3A–F” and EV3D–I”). *GEMC1* and *MCIDAS* OE resulted in increased numbers of P73+GFP+ and FOXJ1+GFP+ cells at 4- and 10 dpe (Figs. 3J,K and EV3N,O). To test whether this is sufficient to initiate the differentiation of aRGs to the EC lineage, we examined the expression of S100β (Figs. 3G–I” and EV3J–L”), an established ECs marker and observed elevated numbers of S100β+GFP+ cells at 4- and 10 dpe (Figs. 3L and  EV3P). In summary, *GEMC1* and *MCIDAS* OE in haRGs led to activation of the multiciliated cell differentiation pathway and ECs generation already in 4 dpe, indicating an instant switch of their fate commitment.

**Figure 4:**
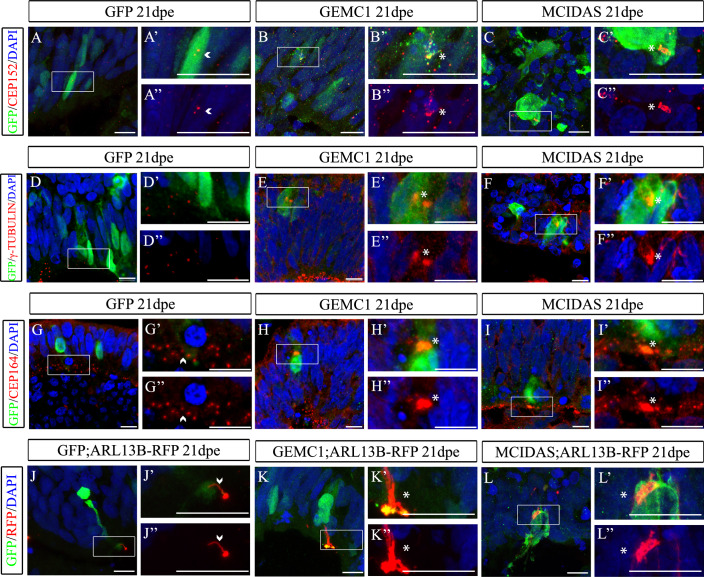
GEMC1 and MCIDAS OE cells exhibit characteristics of mature ECs. (**A**–**I”**) GFP, *GEMC1* or *MCIDAS* OE BOs at 21 dpe stained using antibodies against GFP and CEP152 (**A**–**C”**), γ-TUBULIN (**D**–**F”**) or CEP164 (**G**–**I”**). Biological replicates: CEP152: v = 2 for all conditions. γ-TUBULIN: GFP: v = 2, GEMC1: v = 3, MCIDAS: v = 2. CEP164: GFP: v = 2, GEMC1: v = 1, MCIDAS: v = 2. (**J**–**L”**) BOs were co-transfected with a GFP, *GEMC1* or *MCIDAS* plasmid and an ARL13B-RFP plasmid and at 21 dpe were stained using antibodies against GFP and RFP. Biological replicates: RFP: GFP: v = 2, GEMC1: v = 1, MCIDAS: v = 1. White boxes indicate the area zoomed-in in the corresponding pictures. Arrowheads indicate cells that have two dots of CEP152, γ-TUBULIN, CEP164, or one cilium, and asterisks indicate electroporated cells that exhibit accumulation of CEP152, γ-TUBULIN, CEP164, or ARL13B. Scale bars: 10 μm. Source data are available online for this figure.

**Figure EV4:**
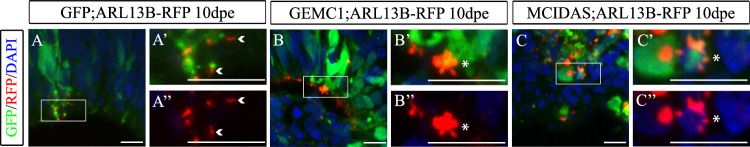
GEMC1 and MCIDAS OE cells showcase multiple cilia. (**A**–**C”**) BOs co-transfected with a GFP, GEMC1 or MCIDAS plasmid and an ARL13B-RFP plasmid at 10 dpe were stained using antibodies against GFP and RFP. Biological replicates: v = 2 for all conditions. White boxes indicate the area zoomed-in in the corresponding pictures. Arrowheads indicate cells that have one cilium and asterisks indicate electroporated cells that exhibit accumulation of ARL13B. Scale bars: 10 μm.

EC maturation is completed ~3 weeks after the commitment of aRGs, as multiple basal bodies and cilia are produced after birth in mice (Spassky et al, 2005). This process is much faster after ectopic OE of either of the genes as it takes ~5–6 days (Kyrousi et al, 2015). We thus questioned whether the S100β+ ECs we observed in *GEMC1* and *MCIDAS* OE BOs acquire their final characteristics. We examined whether they proceeded with the multiplication of their basal bodies and cilia at 21 dpe. At the *GEMC1* and *MCIDAS* OE BOs, we observed an accumulation of clusters of CEP152+ (Fig. 4A–C”), γ-TUBULIN+ (Fig. 4D–F”) and CEP164+ (Fig. 4G–I”) basal bodies, a characteristic associated with mature ECs. To check if manipulated cells have multiple cilia, we co-electroporated *GEMC1*, *MCIDAS* or GFP together with a plasmid expressing ARL13B-RFP (Figs. 4J–L” and EV4A–C”). OE of *GEMC1* and *MCIDAS* resulted in the accumulation of ARL13B at 10- (Fig. EV4A–C”) and at 21 dpe (Fig. 4J–L”), indicating the presence of clusters of cilia and thus EC maturation of the OE cells. Summarizing, our data indicate that upon *GEMC1* and *MCIDAS* OE haRGs lose their identity and cellular morphology and acquire characteristics of mature ECs, highlighting the effect of the two proteins on hEC fate acquisition and differentiation. In addition, our data may indicate that human ependymogenesis abides by a different maturation time course compared to mice, as even 21 dpe after *GEMC1* and *MCIDAS* OE in hBOs, not all ECs develop multiple cilia, suggesting that they might require a longer time to fully mature. GEMC1/MCIDAS-induced ECs have been shown to be functional in mice (Kaplani et al, 2024). In our human-specific model, we did not examine cilia motility, so additional experiments are necessary for the better characterization of the ectopically induced hECs, to more clearly highlight possible differences between them and endogenous hECs.

### Premature ECs generation influences the embryonic neuroepithelium to acquire human-specific V-SVZ niche characteristics

**Figure 5:**
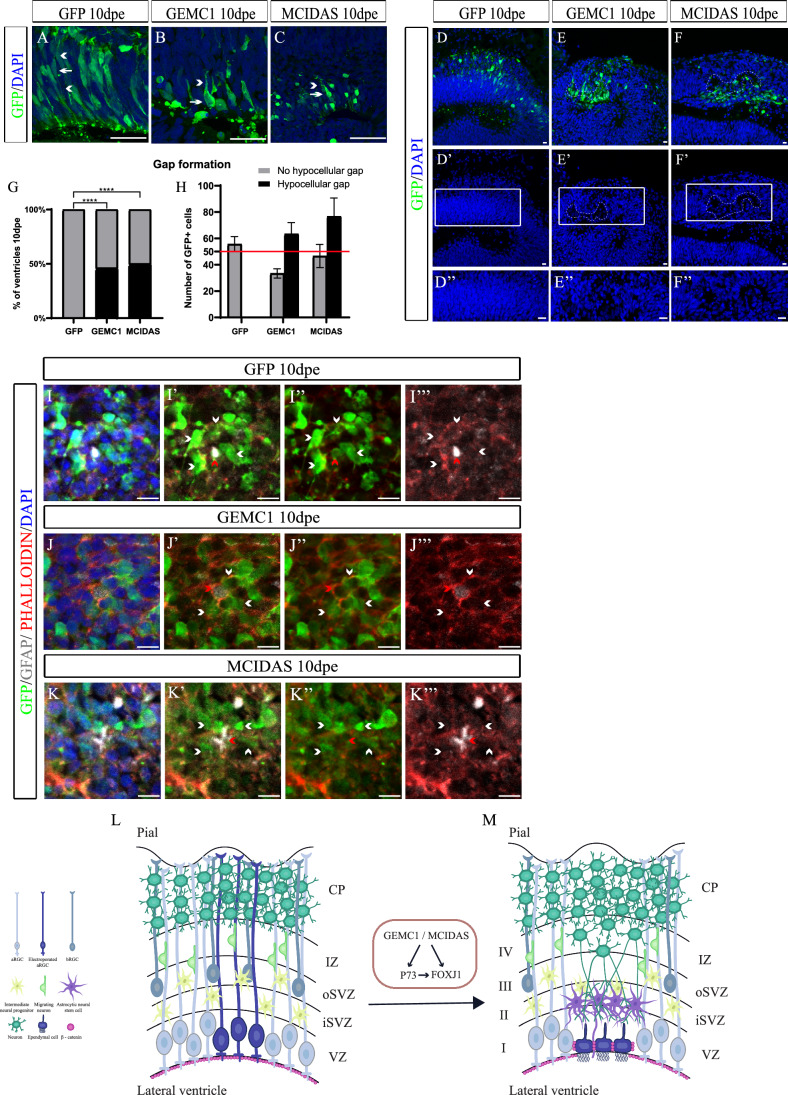
*GEMC1* and *MCIDAS* OE leads the embryonic neural progenitor niche to acquire aV-SVZ niche characteristics. (**A**–**F’**) GFP, *GEMC1* or *MCIDAS* OE BOs at 10 dpe stained using an antibody against GFP. White arrows depict GFP+ cells and white arrowheads the apical and basal processes of the cells. White boxes indicate the area zoomed-in in the corresponding pictures. White lines indicate the borders of the hypocellular gap. (**G**) Quantification of the percentage of ventricles that developed a hypocellular gap (*P* value: <0.0001 and <0.0001, respectively). (**H**) Graph depicting the number of GFP+ cells required to induce the formation of the hypocellular gap. Biological replicates: Gap formation: GFP: v = 23, GEMC1: v = 26, MCIDAS: v = 12. (**I**–**K”’**) GFP, *GEMC1* or *MCIDAS* OE BOs at 10 dpe stained using antibodies against GFP, GFAP, and PHALLOIDIN. Biological replicates: GFP: v = 1, GEMC1: v = 2, MCIDAS: v = 3. White arrowheads indicate GFP+ cells and red arrowheads indicate GFAP+ cells. (**L**, **M**) Cartoon showcasing the cellular organization of a human embryonic V-SVZ niche before (**L**) and after (**M**) GEMC1 and MCIDAS OE. Statistical analysis was performed using the one-tailed exact binomial test (****P < 0.0001). Scale bars (**A**–**F”**): 30 μm, (**I**–**K”**): 10 μm. Source data are available online for this figure.

The ECs that line the lateral ventricles of the adult human brain have characteristic morphological features that distinguish them from the ECs found in mice. More specifically, mouse ECs have a cuboidal shape, whereas hECs bear basal processes that are expanded towards the hypocellular gap of the aV-SVZ niche (Jiménez et al, 2014; Quiñones-Hinojosa et al, 2006). To examine whether the *GEMC1* and *MCIDAS* OE ECs showcase human-specific characteristics, we examined their morphology. We noticed that the majority of the *GEMC1* and *MCIDAS* OE ECs bear a short basal process (Fig. 5A–C), reminiscent of hECs, a phenotype that is not observed in control cells that maintain their radial morphology, nor in the murine aRGs that OE GEMC1 or MCIDAS (Kyrousi et al, 2015). These observations indicate that the ectopically activated GEMC1/MCIDAS pathway in hBOs results in the generation of ECs with human-specific characteristics.

Besides the morphological characteristics of hECs, the haV-SVZ niche has a unique cytoarchitecture that differentiates it from the respective mouse niche. In mice, ECs are adjacent to aNSCs, while hECs are not in contact with them, but instead are separated from them by the hypocellular gap. Interestingly, *GEMC1* and *MCIDAS* OE in BOs resulted in the generation of a gap between the cells that are located next to the ventricular-like structures and the ones that are in a more basal location, as identified by analyzing the distribution of DAPI staining (Fig. 5D–F”), an organization reminiscent of the hypocellular gap. In the manipulated BOs this hypocellular gap is observed in almost 50% of the electroporated ventricular zone-like areas (in 12 *GEMC1* and in 6 *MCIDAS*) while it was not observed in the control condition (Fig. 5G). Moreover, there is positive correlation between the number of OE cells and the formation of the gap, as in small-OE areas (<50 GFP+ cells) ECs are not able to induce a gap formation, whereas in extensively-OE areas (>50 GFP+ cells) a hypocellular gap is observed (Fig. 5H). The *GEMC1* and *MCIDAS* OE cells extend their basal processes towards this newly formed gap (Fig. 5B,C), recapitulating the phenotype observed in adult hECs.

**Figure EV5:**
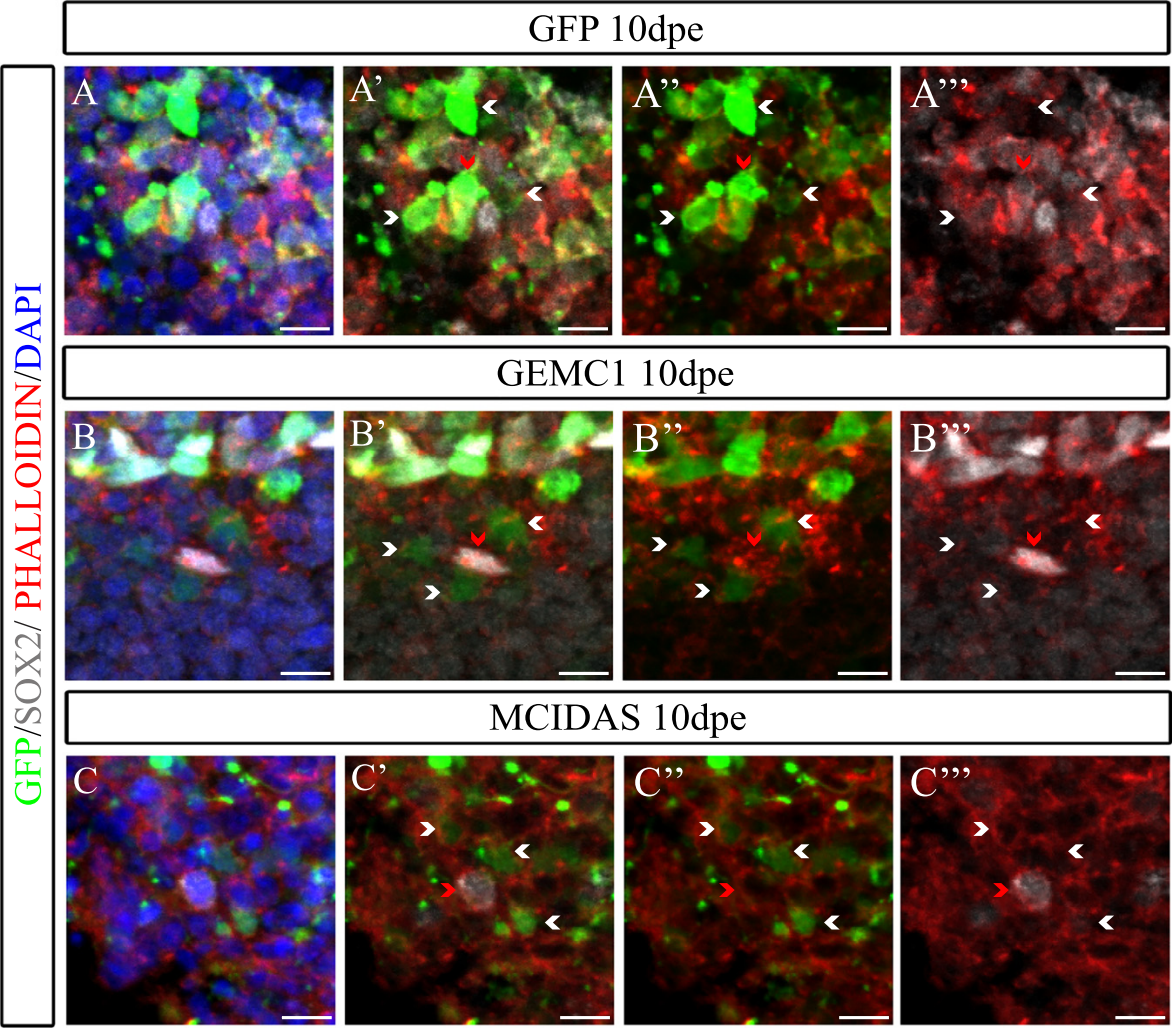
*GEMC1* and *MCIDAS* OE leads to the formation of neural rosettes. (**A**–**C”’**) GFP, *GEMC1* or *MCIDAS* OE BOs at 10 dpe were stained using antibodies against GFP, SOX2 and PHALLOIDIN. Biological replicates: GFP: v = 3, GEMC1: v = 1, MCIDAS: v = 3. White arrowheads indicate GFP+ cells and red arrowheads indicate SOX2+ cells. Scale bars: 10 μm.

It has been shown that ECs alongside aNSCs form characteristic pinwheel structures at the surface of the aV-SVZ niche (Coletti et al, 2018; Mirzadeh et al, 2008). Aiming to investigate whether this phenotype is induced by *GEMC1* and *MCIDAS* OE in hBOs we analyzed thick sections of BOs at 10 dpe using the markers GFAP, for staining aNSCs or astrocytes, or SOX2, for NSCs, alongside PHALLOIDIN (Figs. 5I–K”’ and EV5A–C”’). We were able to detect pinwheel structures where GFP+ OE cells are located around a GFAP+ (Fig. 5J–K”’) or SOX2+ cell (Fig. EV5B–C”’). These data indicate a structural reconfiguration of the niche, highlighting the cell-non-autonomous role of ECs on normal hV-SVZ niche assembly. Similar observations have been reported in mice (Kaplani et al, 2024). We also observed SOX2+GFP+ cells outside of the neural rosettes, as in our PAX6 immunostaining (Figs. 1K–M” and EV1H–J”). These cells could be indicative of incomplete ependymal programming or could represent a transient progenitor of NSCs and ECs. In mice, it has been shown that ECs and aNSCs are generated through a common glial lineage (Fuentealba et al, 2015; Merkle et al, 2004; Spassky et al, 2005). However, whether they share a common endogenous RG ancestor in humans or have different developmental origins remains unclear, and it would be interesting to investigate in a future study. In addition, whether the niche could be neurogenically active and functional remains to be investigated.

The impact of the GEMC1/MCIDAS pathway in the haV-SVZ assembly and EC generation marks them as promising reprogramming factors that could aid in the restoration of normal brain function, as recently suggested in mice (Kaplani et al, 2024). Aberrant aV-SVZ niche formation is linked to disorders such as hydrocephalus, the manifestation of which in humans has been linked to alterations of the cell-to-cell junctions of the aV-SVZ niche cells and ECs dysfunction (Garcia-Bonilla et al, 2021; Ji et al, 2022; Sival et al, 2011; de Wit et al, 2008). Interestingly, mutations in *GEMC1* and *MCIDAS* have been found in patients with congenital hydrocephalus (Lalioti et al, 2019a; Robson et al, 2020), emphasizing their role in haV-SVZ niche formation. Recent data imply that dysregulation of ECs function may also be associated with the manifestation of neurodegenerative diseases (Keryer et al, 2011; Nelles and Hazrati, 2022). So, novel therapeutic approaches aiming at the restoration of normal haV-SVZ niche cytoarchitecture could be of great importance.

Summarizing, we propose that *GEMC1* and *MCIDAS* lead to the premature differentiation of RGs into ECs with human-specific characteristics, while their ectopic expression also results in the reconfiguration of the embryonic neuroepithelium into acquiring haV-SVZ niche characteristics (Fig. 5L,M), highlighting the effect of ECs on the acquisition of the niche. The experimental model we present here can be an excellent tool for the generation of hECs, the study of the haV-SVZ niche assembly and can be used for investigating the pathological manifestations of cilia-related brain disorders or it can further be used to study neurodegenerative disorders.

## Methods

**Table:** Reagents and tools table

Reagent/resource	Reference or source	Identifier or catalog number
**Experimental models**		
Induced pluripotent stem cells reprogrammed from HMGU1 human normal newborn foreskin fibroblasts	Kunze et al, 2018	
**Recombinant DNA**		
pCAGGS-IRES-GFP	Kyrousi et al, 2015	
pCAGGS-IRES-GEMC1-GFP	Kyrousi et al, 2015	
pCAGGS-IRES-MCIDAS-GFP	Kyrousi et al, 2015	
ARL13B-RFP	O’Neill et al, 2018	
**Antibodies**		
Chicken anti-GFP	Aves Lab	GFP-1020
Rabbit anti-Ki-67	Abcam	ab15580
Rabbit anti-Pax6	Biolegend	PRB-278p
Rabbit anti-hMcidas	Pefani et al, 2011	
Rabbit anti-P73	Abcam	ab40658
Mouse anti-Foxj1	eBioscience	14-99-65-80
Mouse anti-S100β	Sigma-Aldrich	S2532
Rabbit anti-Cep152	Bethyl Lab	A302-480A-T
Mouse anti-β-catenin	BD Bioscience	610154
Mouse anti-Map2	Sigma-Aldrich	M4403
Mouse anti-NeuN	Merck Millipore	MAB377
Chicken anti-RFP	Rockland	600-901-379
Anti-Chicken	Life Technologies	A-11039
Alexa Fluor® 568 Goat Anti-Mouse IgG (H + L)	Life Technologies	A-11004
Alexa Fluor® 568 Goat Anti-Mouse IgG1 (γ1)	Life Technologies	A-21124
Alexa Fluor® 647 Goat Anti-Mouse IgG (H + L)	Life Technologies	A-28181
Alexa Fluor® 647 Goat Anti-Rabbit IgG (H + L)	Life Technologies	A-21245
Alexa Fluor® 568 Goat Anti-Rabbit IgG (H + L)	Life Technologies	A-11011
**Oligonucleotides and sequence-based reagents**		
hGEMC1 PCR primers	This study	Table EV1
hMCIDAS PCR primers	Arbi et al, 2016	Table EV1
**Chemicals, enzymes, and other reagents**		
Accutase	Sigma	A6964
ROCK inhibitor Y-27632	StemCell Technology	72304
Matrigel	Corning	CLS354230
Phalloidin 594 conjugated	Thermo Fisher	Α12381
Mowiol	Sigma	81381
DAPI	Sigma	D9542
**Software**		
ImageJ	https://imagej.nih.gov/ij/index.html	
**Other**		
96-well-U bottom plates	Corning	7007
Electroporator	Harvard Apparatus	ECM830

### Cell culture

Induced pluripotent stem cells used were reprogrammed from HMGU1 human normal newborn foreskin fibroblasts (BJ CRL-2522, ATCC) (Kunze et al, 2018). Subjects gave consent for the generation of iPSCs (ISFi001-A). MTA approval for the use of this line of iPSCs was acquired. Established control iPSC lines were maintained in feeder-free conditions and cultured on Matrigel (CLS354230, Corning) coated plates in mTeSR1 basic medium supplemented with 1× mTeSR1 supplement (85850, StemCell Technology) at 37 °C, 5% CO_2_, and ambient oxygen levels, with daily medium changes. Passaging was done by Accutase (A6964, Sigma) treatment at a split ratio of 1:4, while following splitting, culture medium was supplemented with 10 μM ROCK inhibitor Y-27632 (72304, StemCell Technology). For cryopreservation, iPSCs were stored in a freezing medium composed of 40% supplemented mTeSR1 medium, 50% FBS (10437028, Gibco) and 10% DMSO. During thawing, iPSCs were diluted with supplemented mTeSR1 medium, centrifuged, and resuspended in supplemented mTeSR1 medium with the addition of 10 μΜ ROCK inhibitor Y-27632. Karyotypic analyses of the iPSCs were performed to ensure the genomic integrity and stability of the cell line. Immunofluorescent stainings of the pluripotency markers OCT4, SOX2, and NANOG were performed at iPSCs to assess their pluripotency (Kunze et al, 2018).

### Generation of BOs

BOs derived from iPSCs were generated as previously described (Lancaster and Knoblich, 2014) with some modifications. For the generation of BOs iPSCs with passage number 20 to 30 (P20 to P30) were used. The iPSCs were additionally passaged three to four times prior to the initiation of BOs’ generation and were tested negative for mycoplasma contamination. Regarding the generation of BOs, in brief, at day 0, 9000 single cells were plated into low attachment 96-well-U bottom plates in human embryonic stem cell medium [hESCM; DMEM/F12-GlutaMAX (31331093, Gibco) with 20% Knockout Serum Replacement (10828028), 3% FBS (10437028, Gibco), 1% non-essential amino acids (11140050, Gibco), 0.1 mM 2-mercaptoethanol (31350010, Gibco)] supplemented with 4 ng/ml human recombinant FGF [bFGF (100-18B, Gibco)] and 50 µM ROCK inhibitor Y27632 for 4 days to form embryoid bodies (EBs). In the following 2 days, EBs were cultured in hESCM medium without bFGF and ROCK inhibitor. On day 6, the medium was changed to neural induction medium [NIM; DMEM/F12-GlutaMAX supplemented with 1% N_2_ supplement (17502048, Gibco), 1% non-essential amino acids, and 1 µg/ml Heparin (H3149, Sigma)], and EBs were cultured for an additional 6 days. Approximately on day 12 (according to EBs morphology), EBs were embedded in Matrigel droplets and transferred to 10-cm cell culture plates in neural differentiation medium without vitamin A [NDM-A; DMEM/F12-GlutaMAX and Neurobasal (21103049, Gibco) in ratio 1:1 supplemented with 0.5% GlutaMAX supplement (35050038, Gibco), 0.5% N_2_ supplement, 1% B27 supplement without Vitamin A (12587010, Gibco), 0.5% non-essential amino acids, Insulin solution 2.5 µg/ml (I9278, Sigma), 1% antibiotic–antimycotic solution (A5955, Sigma) and 50 µM 2-mercaptoethanol] for 4 days to generate BOs. On day 16, BOs were transferred onto an orbital shaker and cultured in neural differentiation medium plus vitamin A [NDM + A; DMEM/F12-GlutaMAX and Neurobasal in ratio 1:1 supplemented with 0.5% GlutaMAX supplement, 0.5% N_2_ supplement, 1% B27 supplement with Vitamin A (17504044, Gibco), 0.5% non-essential amino acids, insulin solution 2.5 µg/ml, 1% antibiotic–antimycotic solution, and 50 µM 2-mercaptoethanol] at 37 °C, 5% CO_2_, and ambient oxygen levels with medium changes twice a week.

### Plasmid constructs

For the electroporation of BOs mouse cDNAs for *GemC1* and *Mcidas* were cloned in pCAGGS-IRES-GFP vectors between SacI/EcoRV and NheI/SmaI restriction sites, respectively, as previously described (Kyrousi et al, 2015). The ARL13B-RFP plasmid was acquired as previously described (O’Neill et al, 2018).

### Electroporation of organoids

Electroporation of BOs was performed as previously described (Rapti and Kyrousi, 2025). More specifically, a few hours prior to electroporation, organoids were kept in neural differentiation medium without antibiotics. During the procedure, BOs were placed in an electroporation chamber (Harvard Apparatus) under a stereoscope and using a glass microcapillary 1–2 μl of the plasmids IRES-GFP, GEMC1-IRES-GFP or MCIDAS-IRES-GFP alone or in combination with ARL13B-RFP at a final concentration of 1 μg/μl, were injected, together with Fast Green 0.1%, into different ventricles of the brain organoids. The BOs were then subjected to five pulses at 80 V with a 50-ms duration in intervals of 500 ms using an ECM830 electroporation device (Harvard Apparatus). Following electroporation, BOs were kept in antibiotic-free NDM + A media for an additional 24 h, which was then changed into the normal media until fixation. Electroporations were performed in BOs 29–32 days after the initial plating of the cells. BOs were fixed 4, 10, and 21 days post electroporation.

### Immunofluorescence

BOs were analyzed 4, 10, and 21 days after electroporation. They were fixed using 4% PFA, for 1 h at 4 °C, cryo-preserved with 30% sucrose for 16 h and stored at −20 °C in OCT. The BOs were sectioned at 16-µm thickness using a cryotome and stored at −20 °C. For the immunostaining, BOs sections were thawed for 10 min and rehydrated with PBS 1× for 15 min at room temperature. Then, the sections were fixed with 4% PFA for 10 min and washed with PBS 1× three times (3 × 5 min). Afterwards, they were treated with 0.3% Triton X-100 for 5 min and washed with PBS 1x three times (3 × 5 min). Finally, sections were incubated in blocking solution containing PBS 1× with Tween 0.1%, 10% FBS, 3% BSA (A4503, Sigma), for 1 h and then incubated with primary antibodies in blocking solution at 4 °C, overnight. The following day, the sections were washed with PBS 1× with Tween 0.1% three times (3 × 5 min), incubated with secondary antibodies in blocking solution for 1 h at room temperature, washed with PBS 1× with Tween 0.1% three times (3 × 5 min) and with PBS 1× once (1 × 5 min). DNA was stained with DAPI (D9542, Sigma) and the sections were washed with PBS 1× two times (2 × 5 min) and mounted with Mowiol (81381, Sigma). All immunostainings performed for qualitative or quantitative purposes were carried out on at least 2 BOs, as indicated in the Figure Legends. The antibodies that were used for the immunostainings are listed in the Reagents and Tools table.

### En-face imaging of brain organoids

BOs’ ventricular-like areas do not have a specific orientation or position within the tissue, which results in the generation of ventricular-like structures in random places within the BOs, so en-face imaging cannot be performed following a specific protocol, due to difficulties in dissecting the areas properly. In order to address this inquiry, we have sectioned electroporated BOs in thick sections of 30 μm in a cryotome to preserve as much as possible the 3D organization of the electroporated area, and performed immunostainings as previously described.

### RNA isolation and quantitative PCR

Total RNA from hBOs was isolated using the RNeasy Mini extraction kit (74104, Qiagen). cDNA synthesis was performed using the Maxima H Minus Reverse Transcriptase (EP0751, Thermo Fisher) with oligo(dT)16 primers (N8080128, Invitrogen) and random hexamers (51-01-18-25, IDT DNA Technologies) in a 1:1 ratio. Quantitative PCR (RT-qPCR) reactions were run in triplicate using PrimeTime qPCR Primer Assays (IDT DNA Technologies) and PrimeTime® Gene Expression Master Mix (1055770, IDT DNA Technologies) on a LightCycler 480 Instrument II (Roche). Relative gene expression levels were quantified using the relative quantification method and normalized with GADPH as an endogenous control gene. For the qPCR analysis, three independent replicates were included, each bearing the homogenate of three individual BOs. Primer sequences are listed in Table EV1.

### Data analysis

Immunofluorescence images were obtained using a confocal fluorescence microscopy Leica TCS SP8 with a Leica DMi8 microscope. Images were further processed using the Fiji, Adobe Photoshop, and Adobe Illustrator software. Quantifications of control, *GEMC1* and *MCIDAS* OE BOs were carried out on images from tissue sections depicting several independent ventricles, as indicated in the Figure legends. Regarding the en-face imaging analysis, we identified electroporated areas that resembled the expected en-face cellular organization of a V-SVZ area (clusters of GFP+ cells in close proximity to one another) and searched for GFAP+ or SOX2+ cells that were surrounded by GFP+ cells with visible Phalloidin-stained borders that resembled a pinwheel. In each BO many ventricles have been analyzed. Each ventricle (v) analyzed developed independently and is considered a biological replicate of neuroepithelia. Each BO analyzed was developed independently and is considered a biological replicate. Each batch analyzed represents an independent experiment and is considered a biological replicate. Data are represented as mean and standard error of the mean. Normality was tested for each experiment using the Shapiro–Wilk test to determine whether the sample data came from a normally distributed population, and statistical analyses were performed using the appropriate tests. Statistical analysis to show differences in the expression levels of GEMC1 and MCIDAS in brain organoids in different developmental stages (RT-qPCR analysis) was performed using the unpaired *t* test. Statistical analyses to show differences in the percentages of the marker-expressing cells in the different immunofluorescence experiments were performed with the nonparametric two-tailed Mann–Whitney test, while the exact binomial test was used to assess the expected and observed probability of the observed yes or no phenotypes. Differences in the mean values were considered to be significant at *P* < 0.05 (*P < 0.05, **P < 0.01, ***P < 0.001, ****P < 0.0001).

The majority of the study incorporated blinding, and all samples were included without applying specific inclusion or exclusion criteria. The data were critically analyzed by different researchers.

### Adherence to community standards

This study was conducted in accordance with the adapted MDRA checklist provided by *EMBO Reports* in order to ensure methodological transparency and reporting accuracy. In addition, authorship has been determined following the recommendations of the International Committee of Medical Journal Editors (ICMJE), whereby only individuals who made substantial contributions to the conception, design, execution, or interpretation of the research, and who approved the final version of the manuscript, are listed as authors.

## Supplementary information

Table EV1

Peer Review File

Source data Fig. 1

Source data Fig. 2

Source data Fig. 3

Source data Fig. 4

Source data Fig. 5

Expanded View Figures

## Data Availability

This study includes no data deposited in external repositories. The source data of this paper are collected in the following database record: biostudies:S-SCDT-10_1038-S44319-025-00621-3.
