# Supplementary material for: Modelling human adult V-SVZ niche assembly and ependymal cell generation in brain organoids
Source: EMBO Rep. 2025 Nov 5;27(1):31–49. doi: 10.1038/s44319-025-00621-3 (PMC12796355; doi:10.1038/s44319-025-00621-3)
Supplement: Supplementary file 1 — Table EV1 [file 44319_2025_621_MOESM1_ESM.docx]

**Table EV1: Sets of primers used for qPCR analyses**

| **Set of primers** | **Primer sequence** |
| --- | --- |
| hGEMC1 FW | 5’-ACTCGCCAGGTTACACGAAG-3' |
| hGEMC1 RV | 5’-CCCCTTCCTGAATTTTCCAT-3' |
| hMCIDAS FW | 5’-GACGCGCTTGTTGAGAATAA-3’ |
| hMCIDAS RV | 5’-CACGTTCCGCTCCTTGAG-3’ |
| hGAPDH FW | 5′‐AATCCCATCACCATCTTCCAGGA‐3′ |
| hGAPDH RV | 5′‐TGGACTCCACGACGTACTCAG‐3′ |
