# Supplementary figures and images for: Modelling human adult V-SVZ niche assembly and ependymal cell generation in brain organoids
Source: EMBO Rep. 2025 Nov 5;27(1):31–49. doi: 10.1038/s44319-025-00621-3 (PMC12796355; doi:10.1038/s44319-025-00621-3)

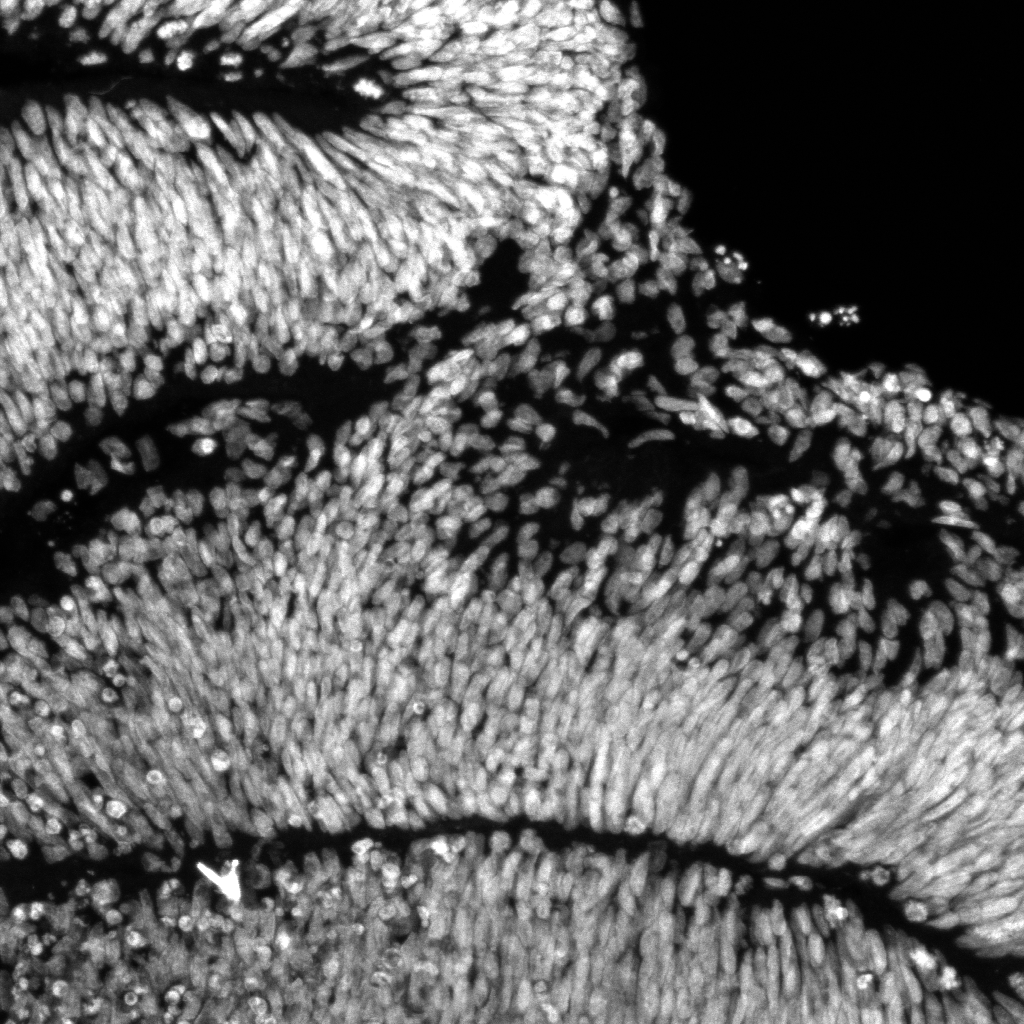

Supplement: Supplementary file 4 — Source data Fig. 2 [file 44319_2025_621_MOESM4_ESM.zip › Figure 2/2D/GFP REELIN 10dpe.tif]

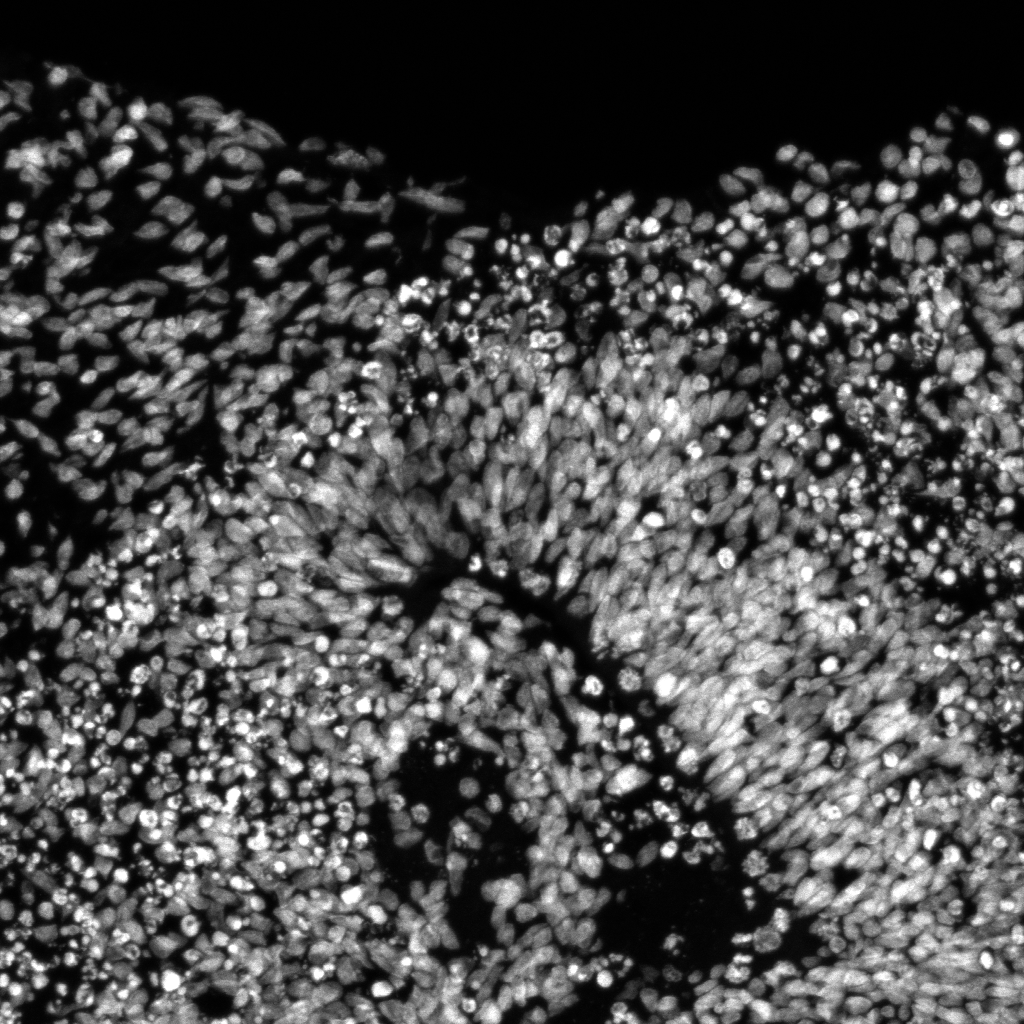

Supplement: Supplementary file 4 — Source data Fig. 2 [file 44319_2025_621_MOESM4_ESM.zip › Figure 2/2E/GEMC1 REELIN 10dpe.tif]

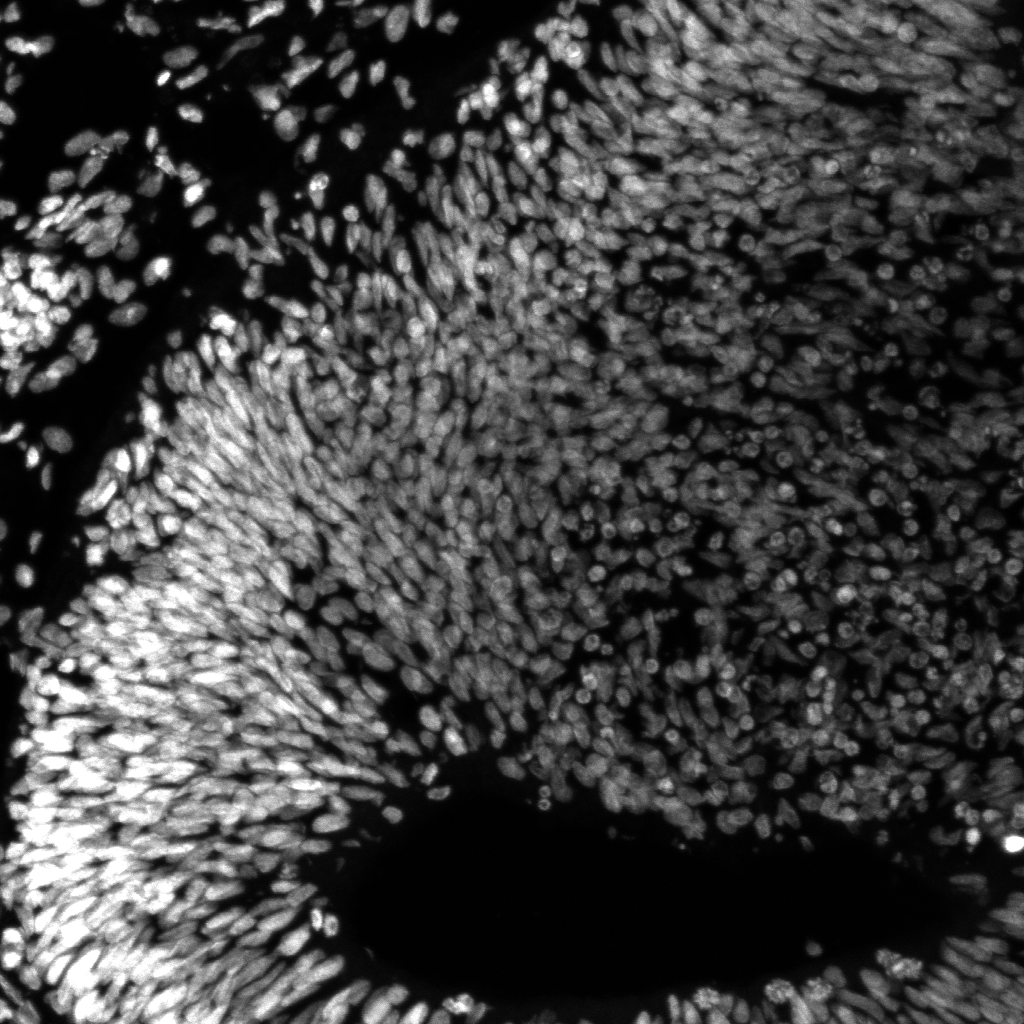

Supplement: Supplementary file 4 — Source data Fig. 2 [file 44319_2025_621_MOESM4_ESM.zip › Figure 2/2F/MCIDAS REELIN 10dpe.tif]

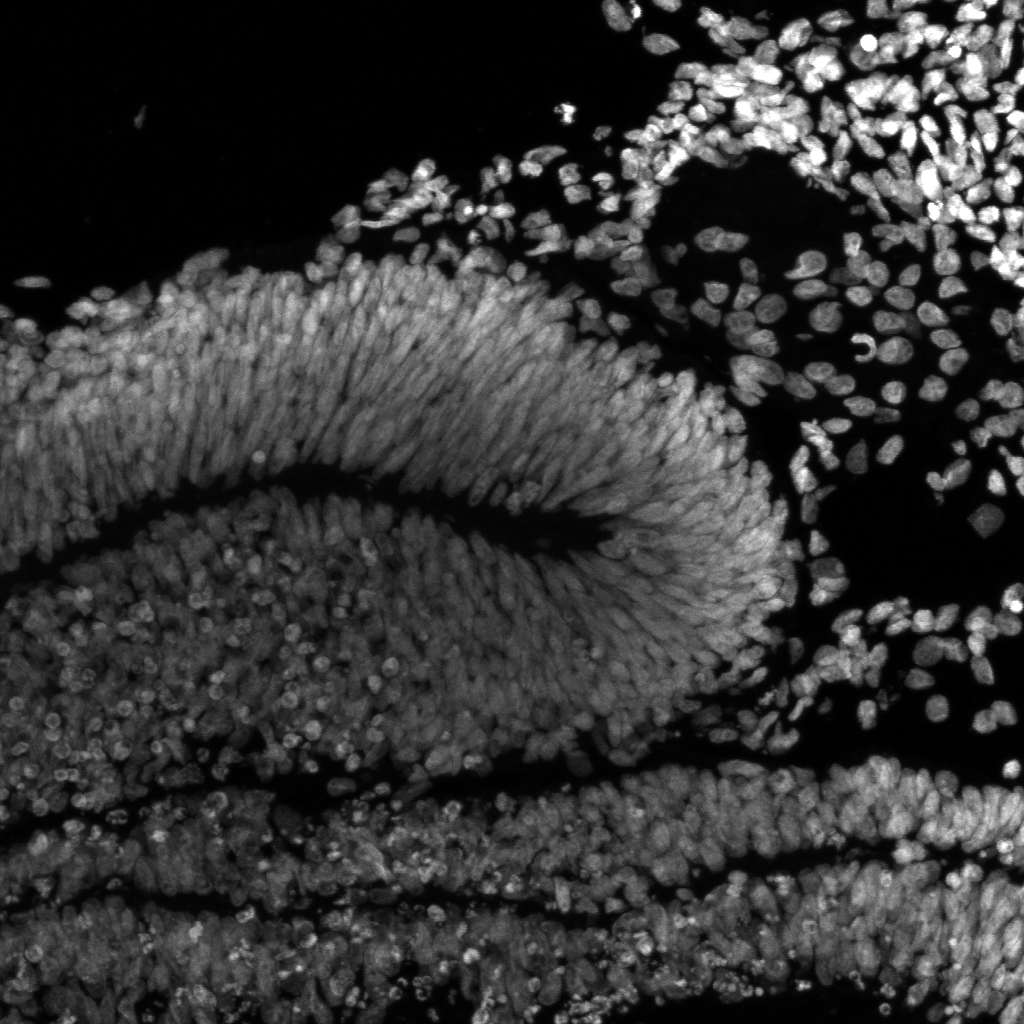

Supplement: Supplementary file 4 — Source data Fig. 2 [file 44319_2025_621_MOESM4_ESM.zip › Figure 2/2G/GFP TTR 10dpe.tif]

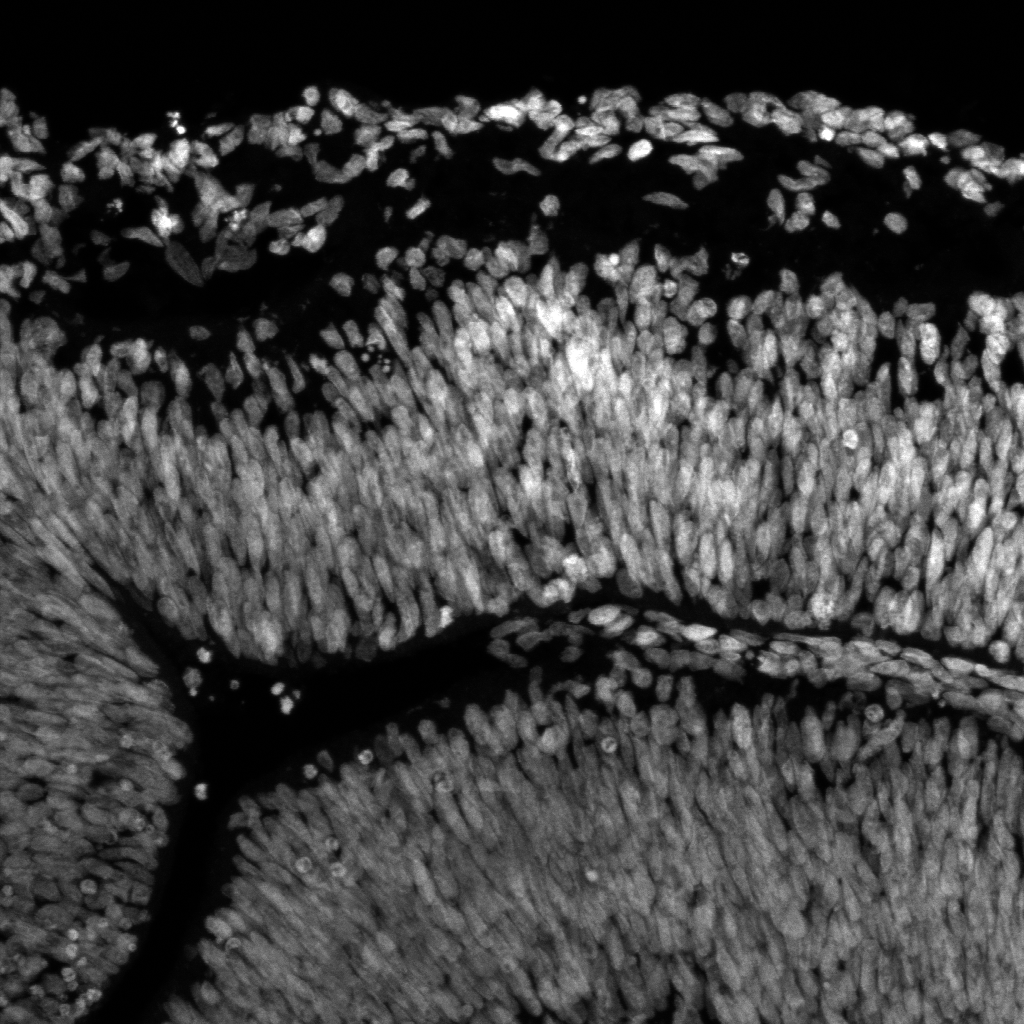

Supplement: Supplementary file 4 — Source data Fig. 2 [file 44319_2025_621_MOESM4_ESM.zip › Figure 2/2H/GEMC1 TTR 10dpe.tif]

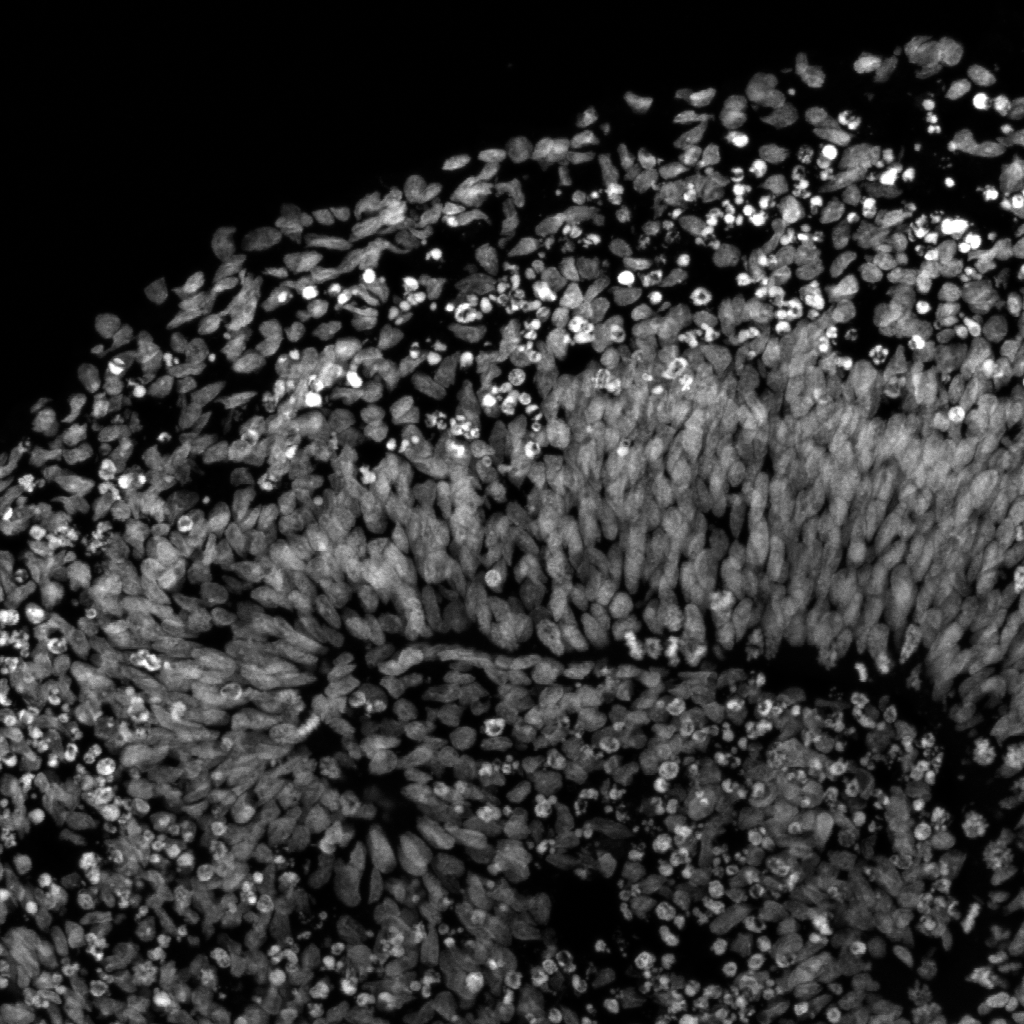

Supplement: Supplementary file 4 — Source data Fig. 2 [file 44319_2025_621_MOESM4_ESM.zip › Figure 2/2I/MCIDAS TTR 10dpe.tif]

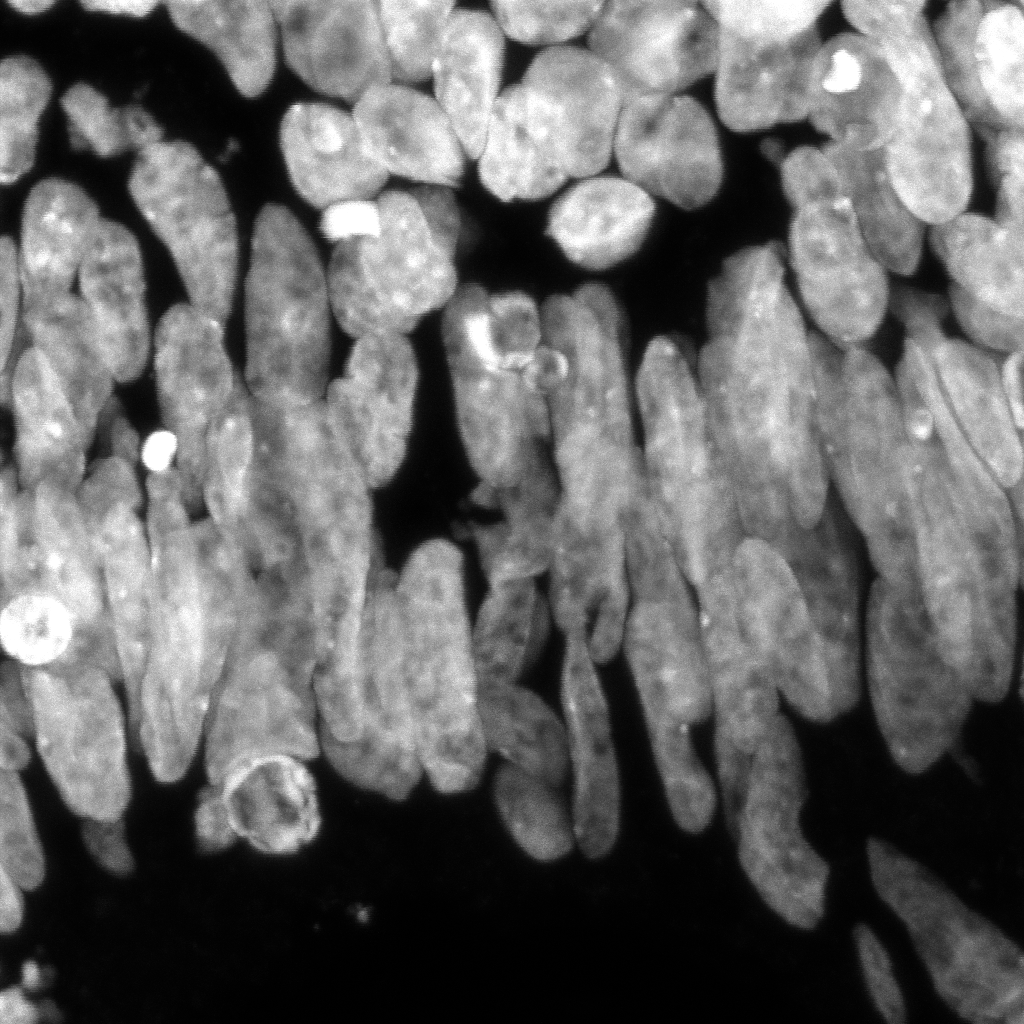

Supplement: Supplementary file 6 — Source data Fig. 4 [file 44319_2025_621_MOESM6_ESM.zip › Figure 4/4D/GFP G-TUBULIN 21dpe.tif]

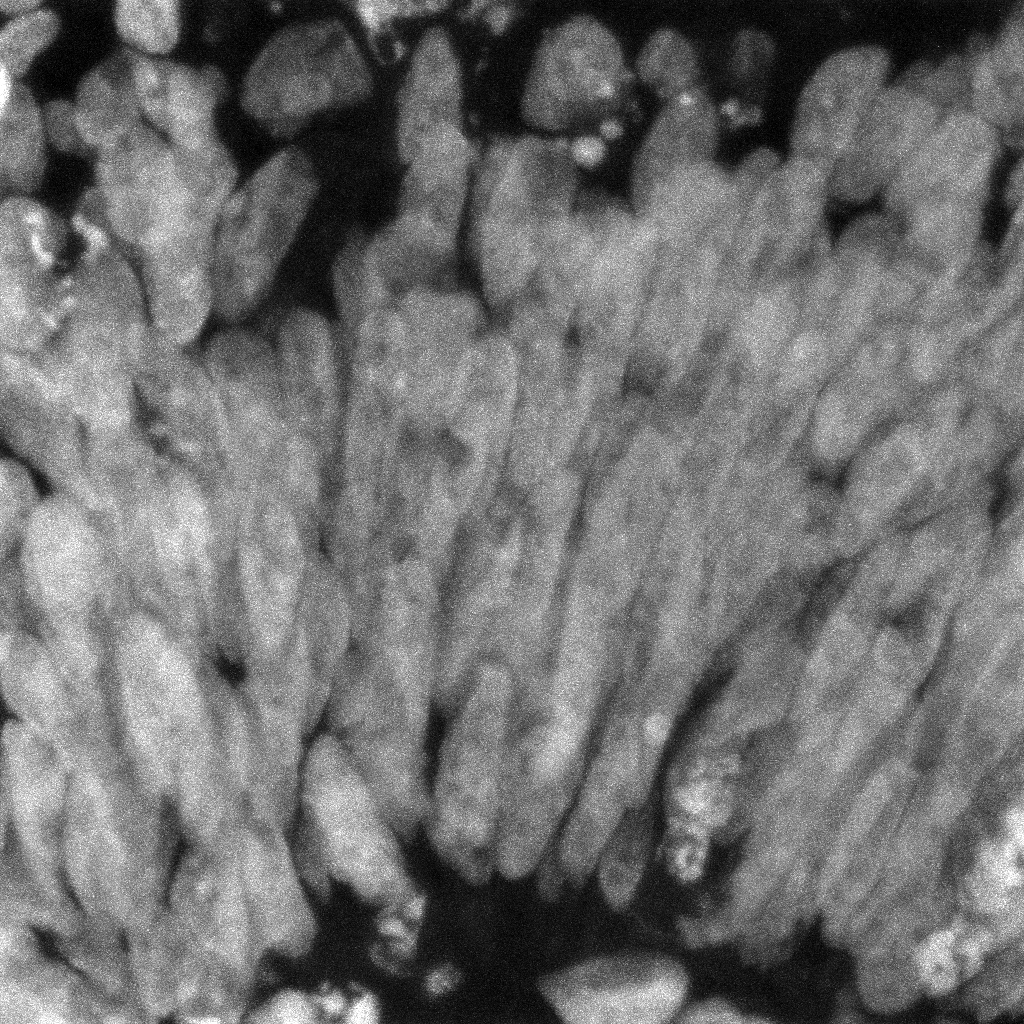

Supplement: Supplementary file 6 — Source data Fig. 4 [file 44319_2025_621_MOESM6_ESM.zip › Figure 4/4E/GEMC1 G-TUBULIN 21dpe.tif]

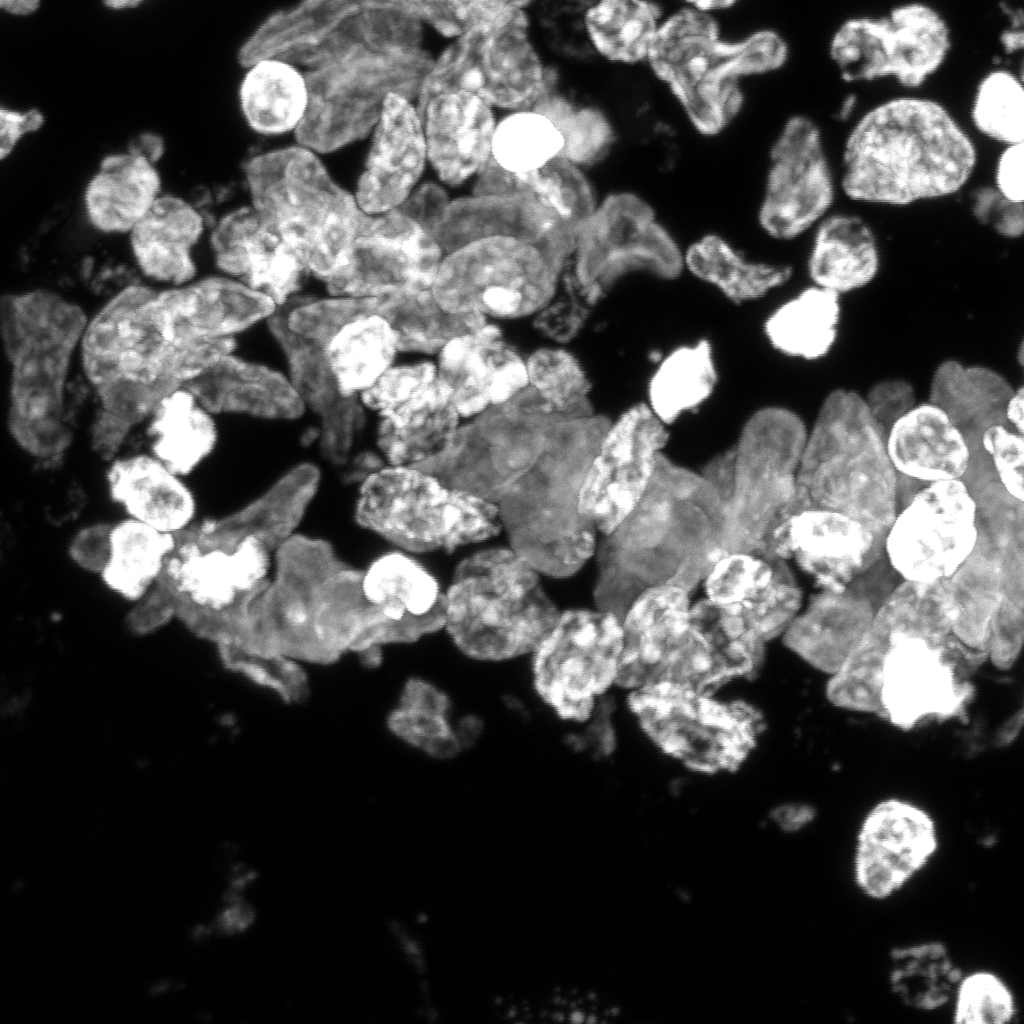

Supplement: Supplementary file 6 — Source data Fig. 4 [file 44319_2025_621_MOESM6_ESM.zip › Figure 4/4F/MCIDAS G-TUBULIN 21dpe.tif]

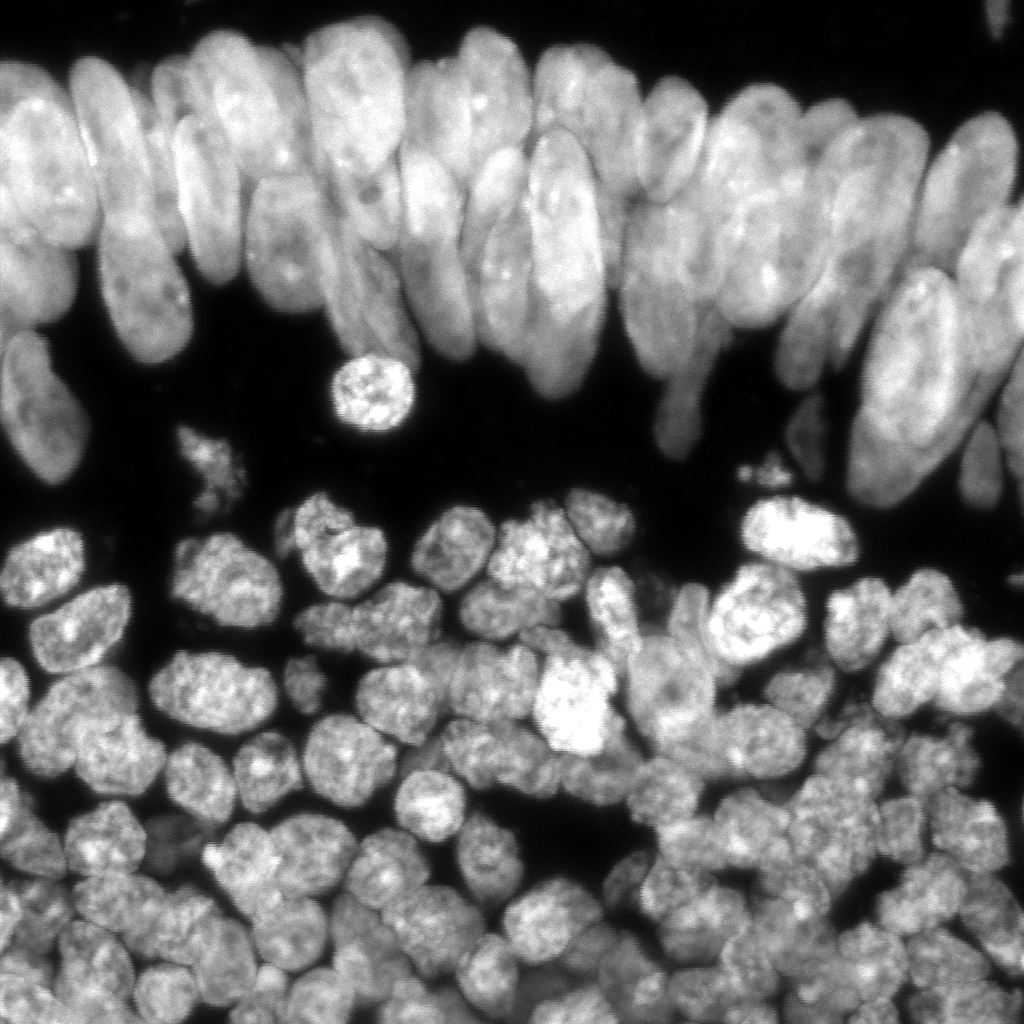

Supplement: Supplementary file 6 — Source data Fig. 4 [file 44319_2025_621_MOESM6_ESM.zip › Figure 4/4G/GFP CEP164 21dpe.tif]

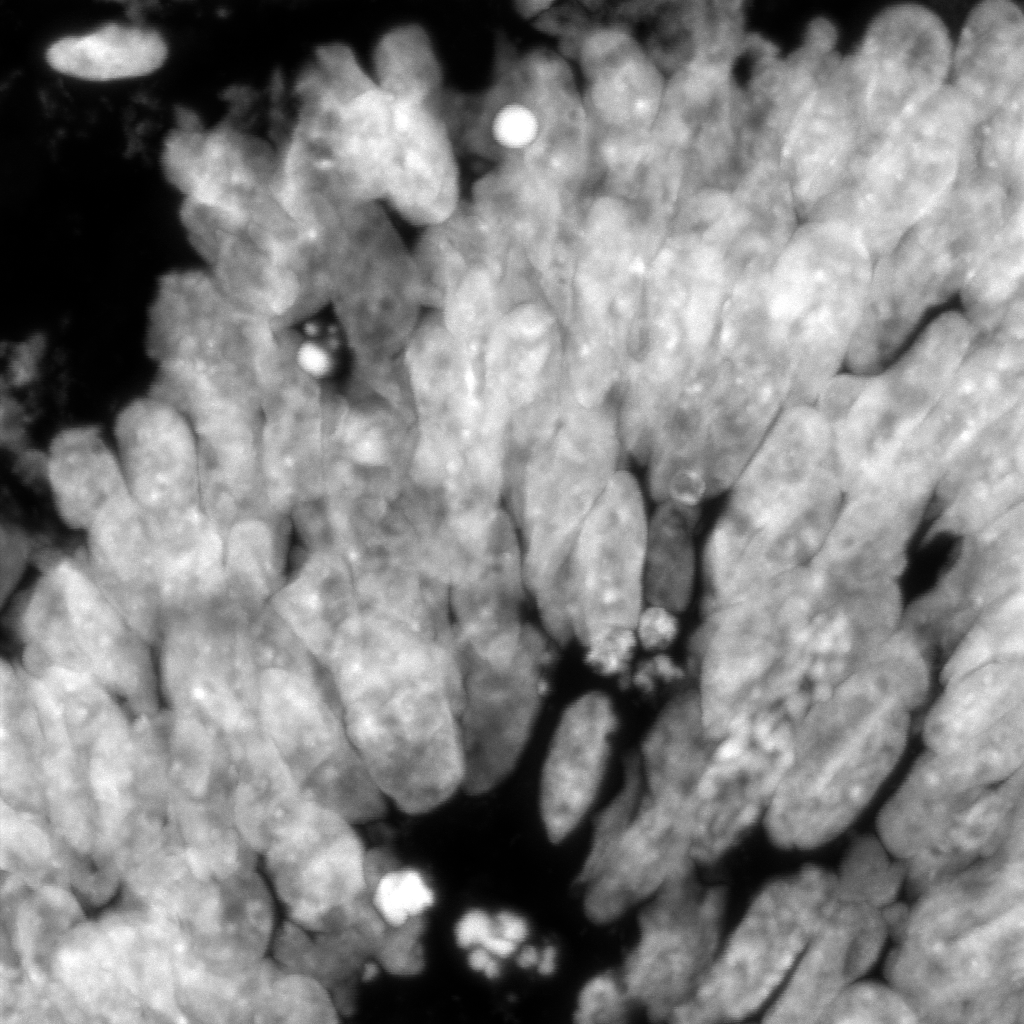

Supplement: Supplementary file 6 — Source data Fig. 4 [file 44319_2025_621_MOESM6_ESM.zip › Figure 4/4H/GEMC1 CEP164 21dpe.tif]

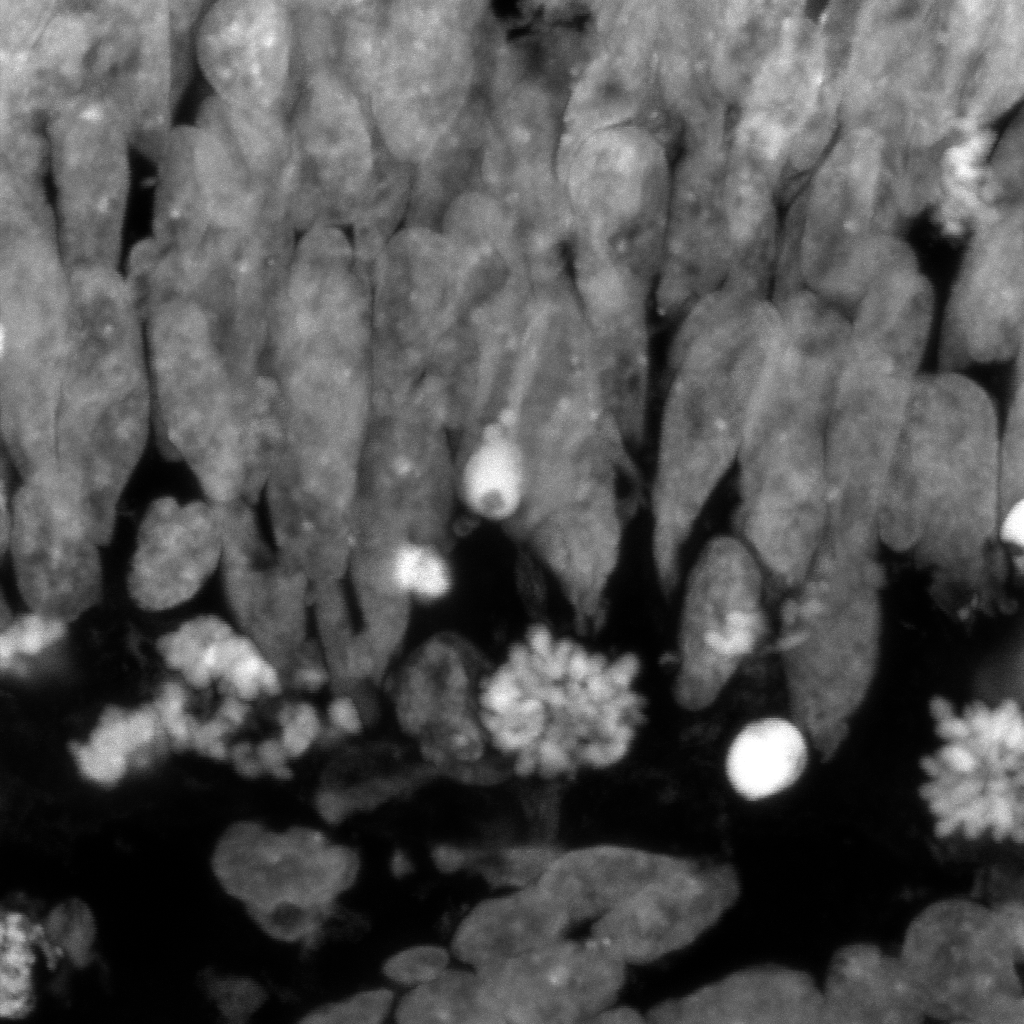

Supplement: Supplementary file 6 — Source data Fig. 4 [file 44319_2025_621_MOESM6_ESM.zip › Figure 4/4I/MCIDAS CEP164 21dpe.tif]

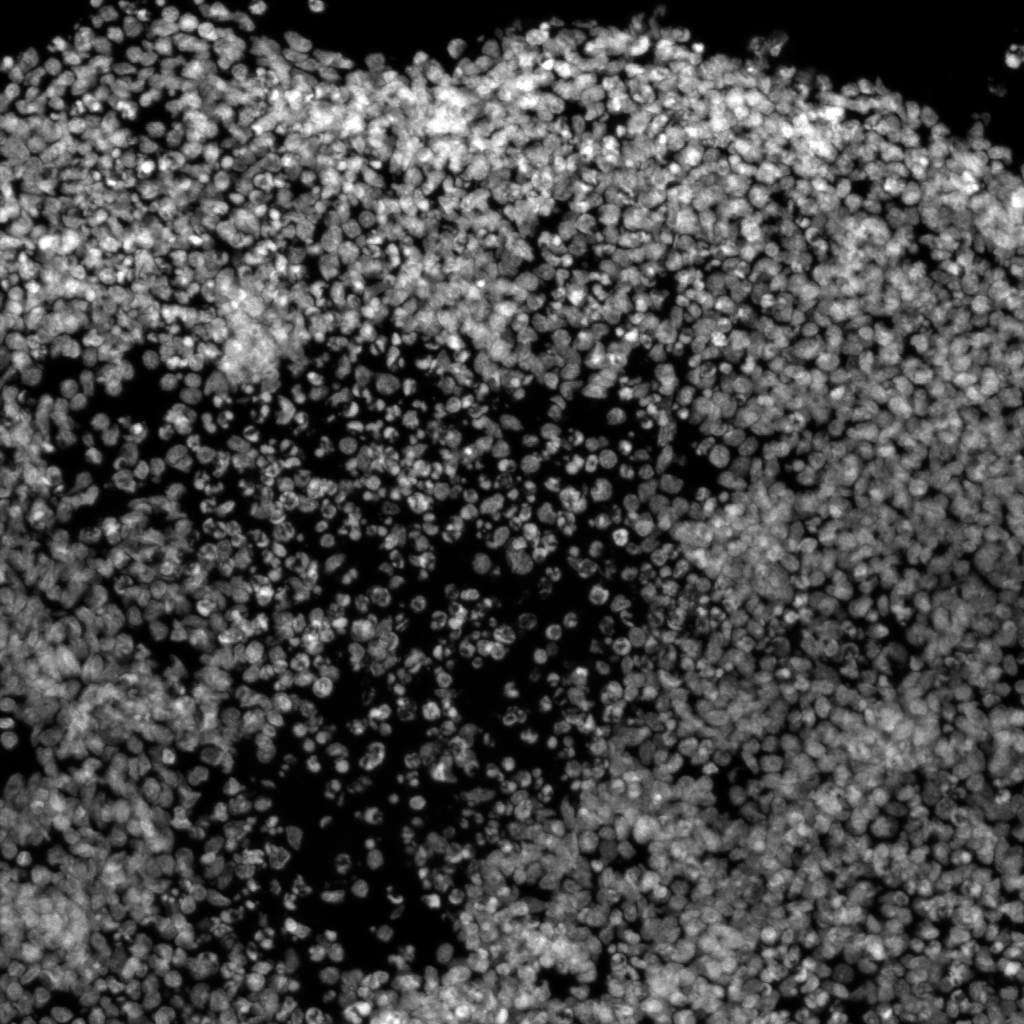

Supplement: Supplementary file 7 — Source data Fig. 5 [file 44319_2025_621_MOESM7_ESM.zip › Figure 5/5I/GFP GFAP PHALLOIDIN en face 10dpe.tif]

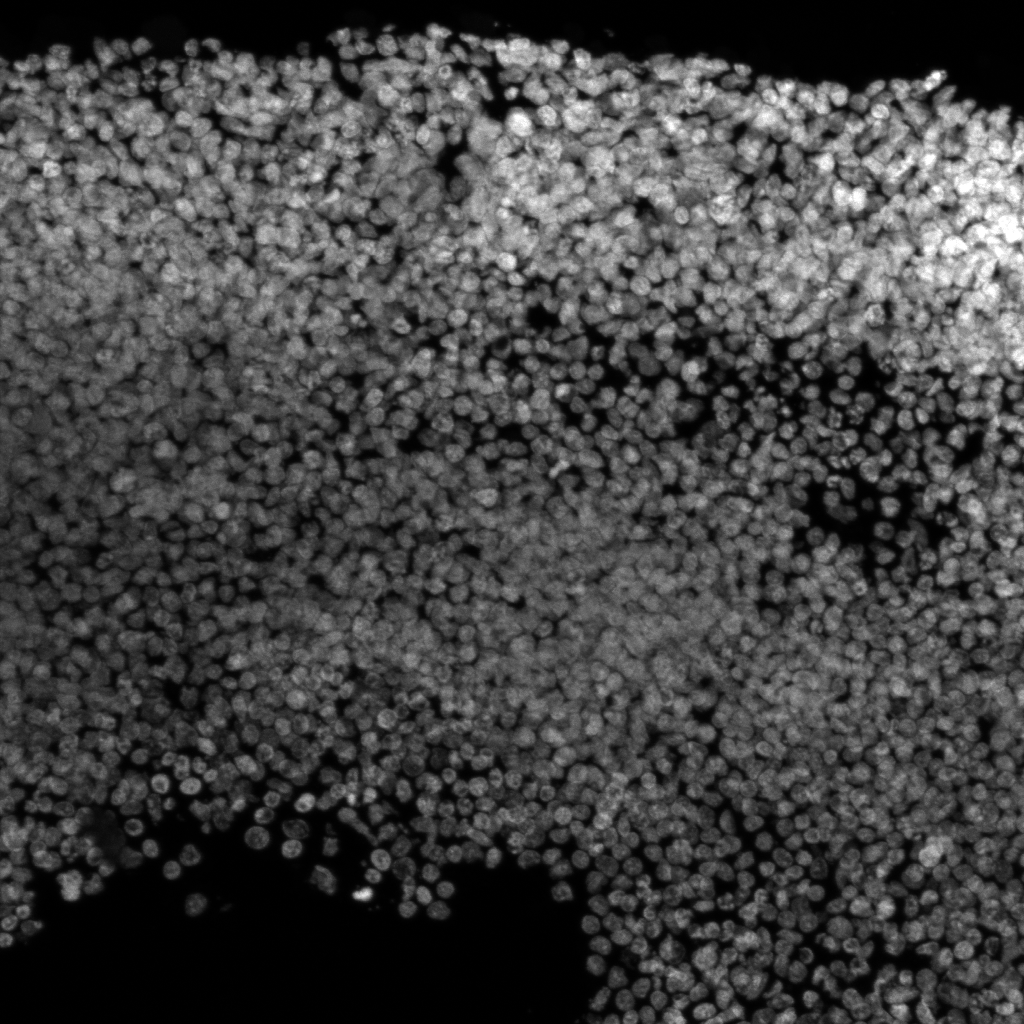

Supplement: Supplementary file 7 — Source data Fig. 5 [file 44319_2025_621_MOESM7_ESM.zip › Figure 5/5J/GEMC1 GFAP PHALLOIDIN en face 10dpe.tif]

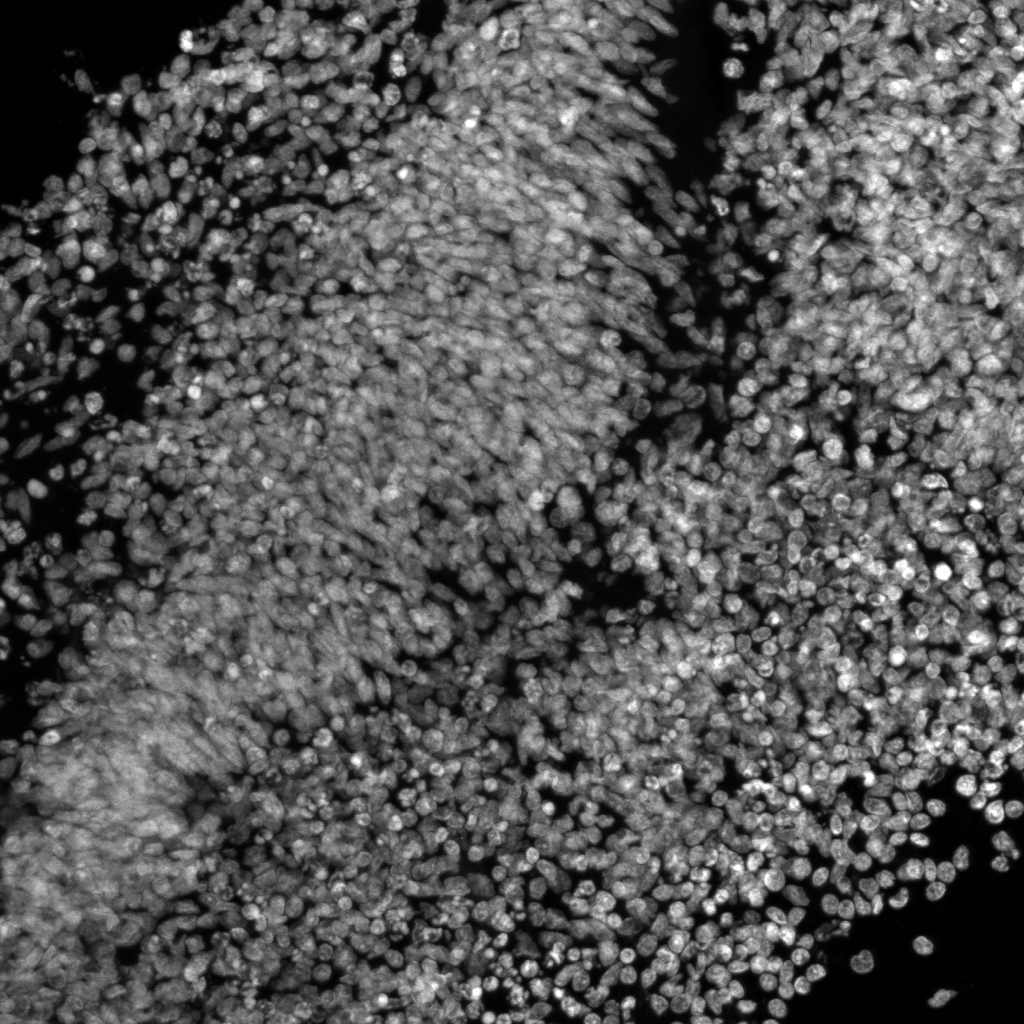

Supplement: Supplementary file 7 — Source data Fig. 5 [file 44319_2025_621_MOESM7_ESM.zip › Figure 5/5K/MCIDAS GFAP PHALLOIDIN en face 10dpe.tif]
