# Supplementary material for: Modelling human adult V-SVZ niche assembly and ependymal cell generation in brain organoids
Source: EMBO Rep. 2025 Nov 5;27(1):31–49. doi: 10.1038/s44319-025-00621-3 (PMC12796355; doi:10.1038/s44319-025-00621-3)
Supplement: Supplementary file 8 — Expanded View Figures [file 44319_2025_621_MOESM8_ESM.pdf]

# Expanded View Figures

**Figure EV1. Cell- and time-specific expression patterns of GEMC1 and MCIDAS and their influence on aRGs number.**

(**A–B′**) Analyses of published single-cell RNA sequencing data in cells isolated from hBOs in different developmental stages (Data ref: Uzquiano et al, 2022). Violin plots showing the GEMC1 and MCIDAS normalized counts at different cell types in 2 m (**A**, **B**) and 6 m hBOs (**A′**, **B′**). (**C**, **D**) GEMC1 and MCIDAS quantitative mRNA expression analyses in hBOs in different developmental stages. Data normalized against GADPH. Three independent replicates were analyzed, each bearing the homogenate of three individual brain organoids. (**E–J″**) GFP, *GEMC1* or *MCIDAS* OE BOs at 4 dpe stained using antibodies against GFP and Ki-67 (**E–G″**) or PAX6 (**H–J**″). Data are represented as the mean ± s.e.m. Statistical analysis was performed using the unpaired *t* test (**P* < 0.05, ****P* < 0.001, *****P* < 0.0001). White boxes indicate the area zoomed-in in the corresponding pictures. Arrows indicate Ki-67+GFP+ or PAX6+GFP+ cells. Scale bars: 30 µm.


© The Author(s)

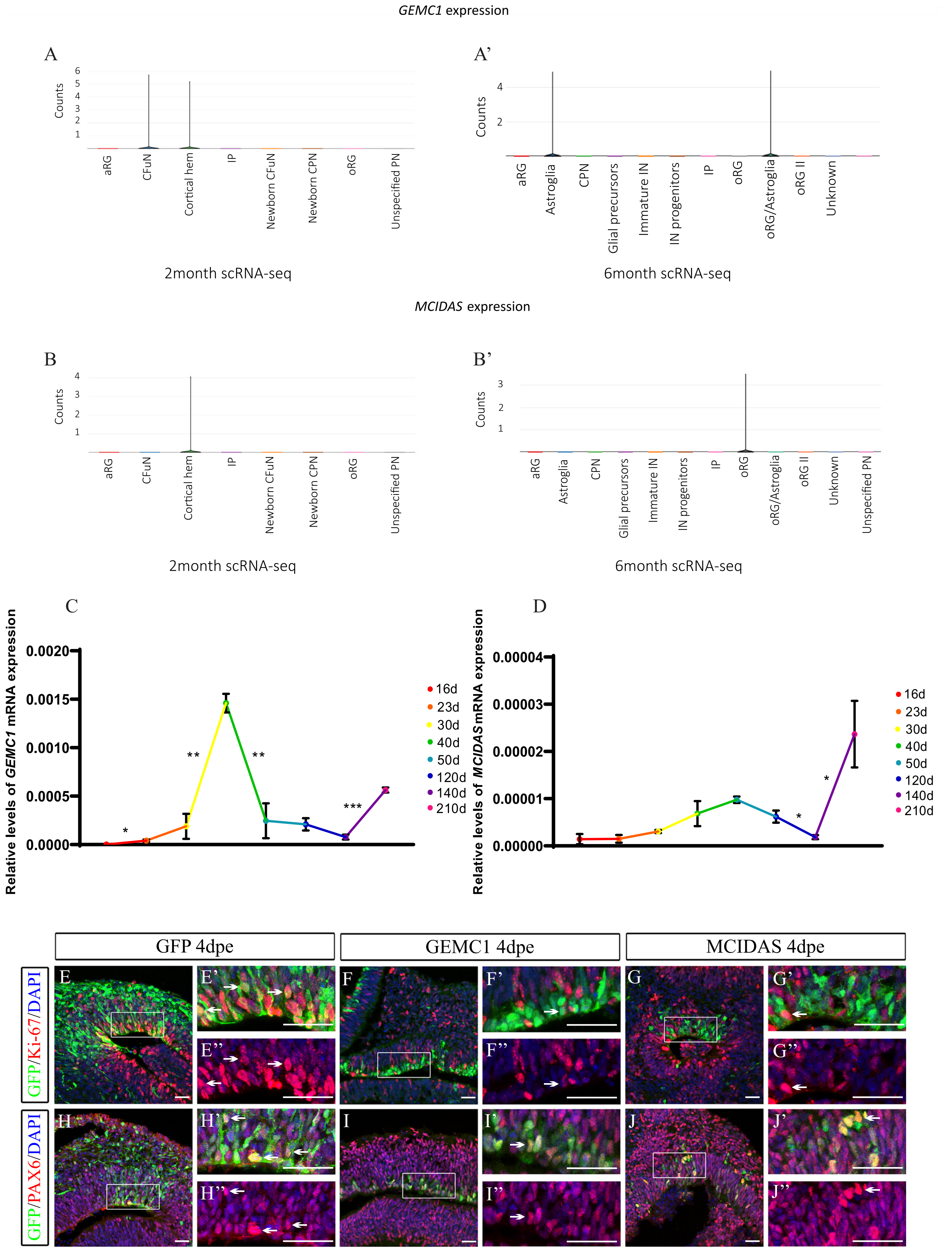
GEMC1 expression
A
Counts
aRG
CFuN
Cortical hem
IP
Newborn CFuN
Newborn CPN
oRG
Unspecified PN
2month scRNA-seq
A'
Counts
aRG
Astroglia
CPN
Glial precursors
Immature IN
IN progenitors
IP
oRG
oRG/Astroglia
oRG II
Unknown
6month scRNA-seq
MCIDAS expression
B
Counts
aRG
CFuN
Cortical hem
IP
Newborn CFuN
Newborn CPN
oRG
Unspecified PN
2month scRNA-seq
B'
Counts
aRG
Astroglia
CPN
Glial precursors
Immature IN
IN progenitors
IP
oRG
oRG/Astroglia
oRG II
Unknown
Unspecified PN
6month scRNA-seq
C
Relative levels of GEMC1 mRNA expression
0.0020
0.0015
0.0010
0.0005
0.0000
16d
23d
30d
40d
50d
120d
140d
210d
D
Relative levels of MCIDAS mRNA expression
0.00004
0.00003
0.00002
0.00001
0.00000
GFP 4dpe
GEMC1 4dpe
MCIDAS 4dpe
GFP/Ki-67/DAPI
GFP/PAX6/DAPI
E
E'
E''
F
F'
F''
G
G'
G''
H
H'
H''
I
I'
I''
J
J'
J''

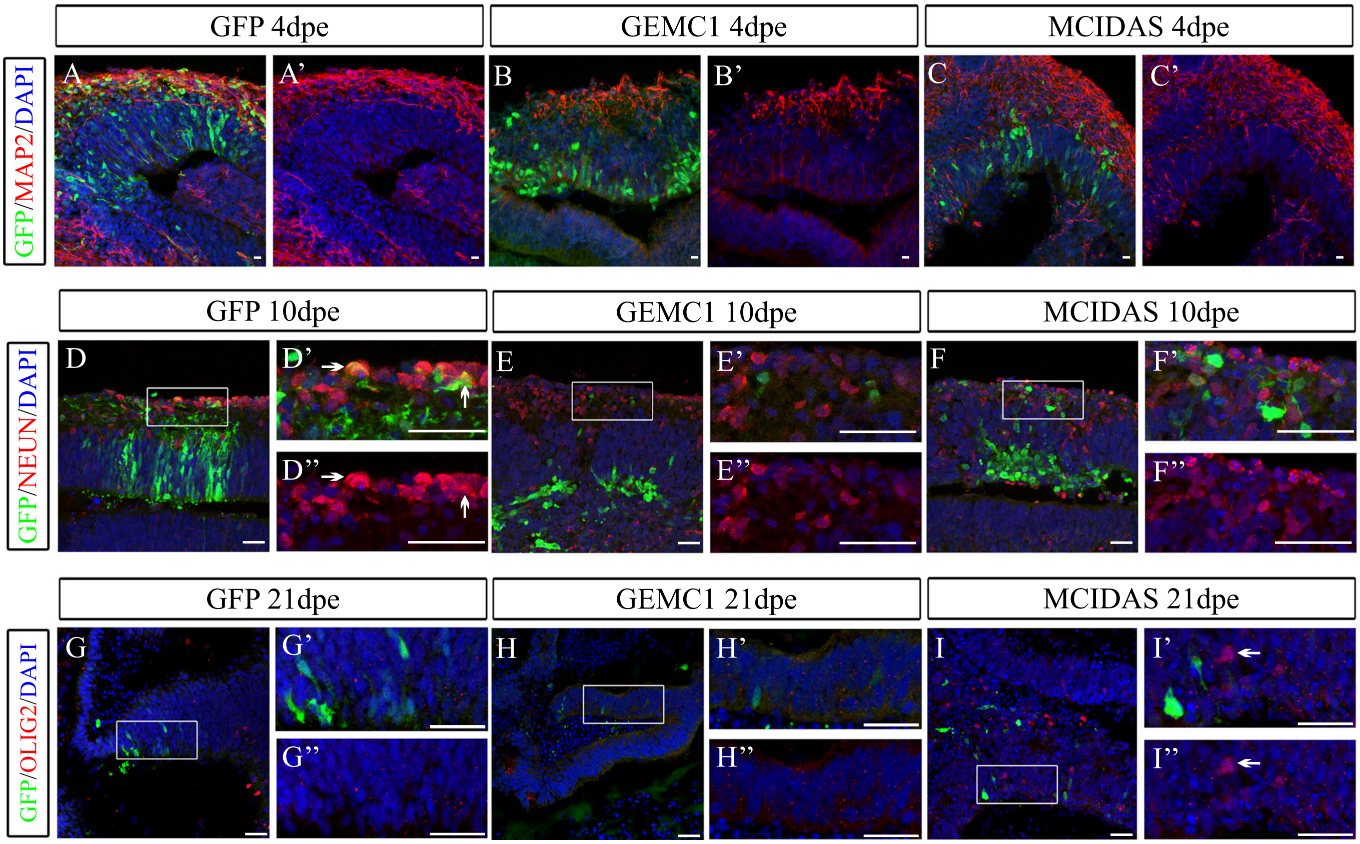


**Figure EV2. GEMC1 and MCIDAS OE cells fail to differentiate into neurons.**

(**A–C′**) GFP, *GEMC1* or *MCIDAS* OE BOs at 4 dpe stained using antibodies against GFP and MAP2. (**D–F″**) GFP, *GEMC1* or *MCIDAS* OE BOs at 10 dpe stained using antibodies against GFP and NEUN. (**G–I″**) GFP, *GEMC1* or *MCIDAS* OE BOs at 21 dpe stained using antibodies against GFP and OLIG2. Biological replicates: MAP2: GFP: v = 9, GEMC1: v = 4, MCIDAS: v = 4. NEUN: v = 3 for all conditions. OLIG2: GFP: v = 1, GEMCI: v = 2, MCIDAS: v = 2. White and red boxes indicate the area zoomed-in in the corresponding pictures. Arrows indicate NEUN+GFP+ or OLIG2+ cells. Scale bars: 30 μm.

 © The Author(s)

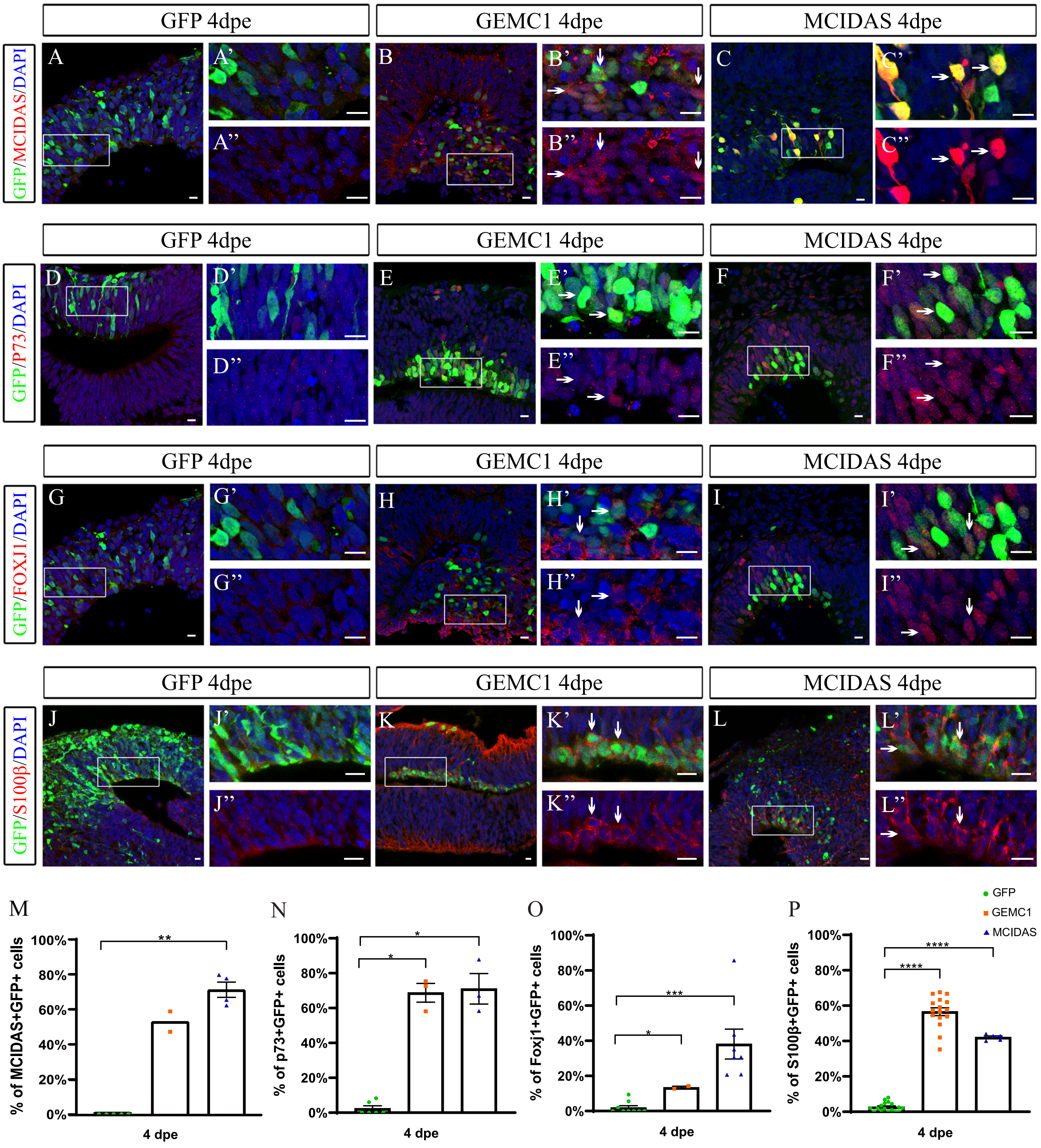


◀ **Figure EV3. GEMC1 and MCIDAS OE cells express ECs markers.**

(**A–L″**) GFP, *GEMC1* or *MCIDAS* OE BOs at 4 dpe stained using antibodies against GFP and MCIDAS (**A–C″**), P73 (**D–F″**), FOXJ1 (**G–I″**) or S100β (**J–L″**). DAPI and GFP channels in images (**A–A″**) are reused in (**G–G″**), DAPI and GFP channels in images (**B–B″**) are reused in (**H–H″**) and DAPI and GFP channels in images (**F–F″**) are reused in (**I–I″**). (**M–P**) Quantification of the percentage of MCIDAS+GFP+ (**M**), P73+GFP+ (**N**), FOXJ1+GFP+ (**O**) and S100β+GFP+ cells (**P**). Biological replicates: MCIDAS: GFP: v = 5, GEMC1: v = 2, MCIDAS: v = 4 (*P* value: 0.0079). P73: GFP: v = 6, GEMC1: v = 3, MCIDAS: v = 3 (*P* value: 0.0119 and 0.0119, respectively). FOXJ1: GFP: v = 9, GEMC1: v = 2, MCIDAS: v = 7 (*P* value: 0.0182 and 0.0002, respectively). S100β: GFP: v = 17, GEMC1: v = 16, MCIDAS: v = 5 (*P* value: <0.0001 and <0.0001, respectively). Data are represented as the mean ± s.e.m. Statistical analysis was performed using the nonparametric two-tailed Mann–Whitney test (**P* < 0.05, ***P* < 0.01, ****P* < 0.001, *****P* < 0.0001). White boxes indicate the area zoomed-in in the corresponding pictures. Arrows indicate MCIDAS+GFP+, P73+GFP+ cells, FOXJ1+GFP+ cells or S100β+GFP+ cells. Scale bars: 10 μm.

© The Author(s)

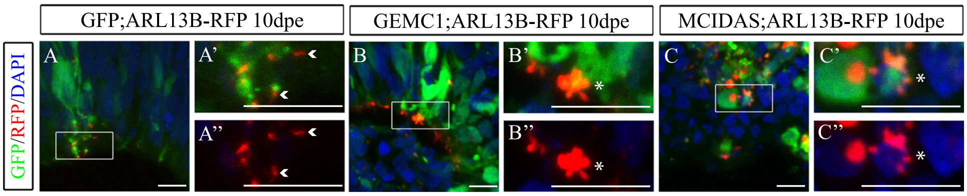


**Figure EV4. GEMC1 and MCIDAS OE cells showcase multiple cilia.**

**(A–C″)** BOs co-transfected with a GFP, GEMC1 or MCIDAS plasmid and an ARL13B-RFP plasmid at 10 dpe were stained using antibodies against GFP and RFP. Biological replicates: v = 2 for all conditions. White boxes indicate the area zoomed-in in the corresponding pictures. Arrowheads indicate cells that have one cilium and asterisks indicate electroporated cells that exhibit accumulation of ARL13B. Scale bars: 10 µm.

 © The Author(s)

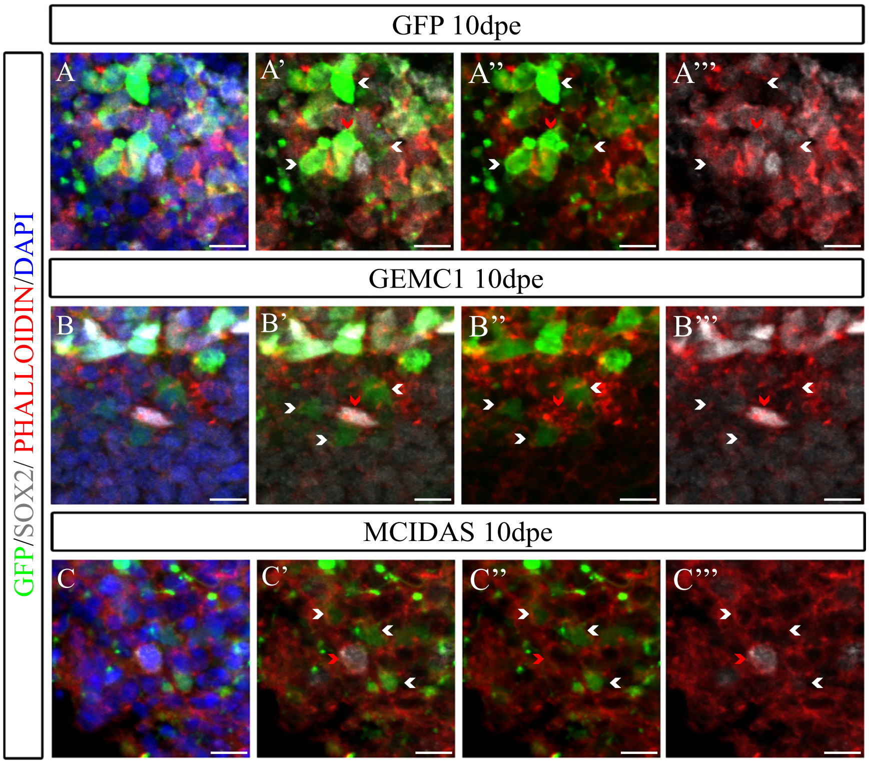


**Figure EV5. *GEMC1* and *MCIDAS* OE leads to the formation of neural rosettes.**

**(A–C‴)** GFP, *GEMC1* or *MCIDAS* OE BOs at 10 dpe were stained using antibodies against GFP, SOX2 and PHALLOIDIN. Biological replicates: GFP: ν = 3, GEMC1: ν = 1, MCIDAS: ν = 3. White arrowheads indicate GFP+ cells and red arrowheads indicate SOX2+ cells. Scale bars: 10 µm.
