# Supplementary material for: Modelling human adult V-SVZ niche assembly and ependymal cell generation in brain organoids
Source: EMBO Rep. 2025 Nov 5;27(1):31–49. doi: 10.1038/s44319-025-00621-3 (PMC12796355; doi:10.1038/s44319-025-00621-3)
Supplement: Supplementary file 2 — Peer Review File [file 44319_2025_621_MOESM2_ESM.pdf]

EMBO reports

Modelling human adult V-SVZ niche assembly and ependymal cell generation in brain organoids

Styliani Vassalou, Maria-Eleni Lalioti, Rossella Di Giaimo, Stavros Taraviras, Silvia Cappello, and Christina Kyrousi

Corresponding author(s): Christina Kyrousi (ckyrousi@med.uoa.gr) , Stavros Taraviras (taraviras@med.upatras.gr), Silvia Cappello (silvia.cappello@bmc.med.lmu.de)

Review Timeline:

Submission Date:	17th Feb 25
Editorial Decision:	8th Apr 25
Revision Received:	9th Sep 25
Editorial Decision:	8th Oct 25
Revision Received:	15th Oct 25
Accepted:	23rd Oct 25

Editor: Esther Schnapp

Transaction Report:

(Note: With the exception of the correction of typographical or spelling errors that could be a source of ambiguity, letters and reports are not edited. Depending on transfer agreements, referee reports obtained elsewhere may or may not be included in this compilation. Referee reports are anonymous unless the Referee chooses to sign their reports.)

1st Editorial Decision **8th Apr 2025**

Dear Christina,

Thank you for the submission of your manuscript to EMBO reports. It was sent to 3 referees but we have only received the comments from 2 of them, despite sending several reminders. Given that both referees are in fair agreement, I am making a decision now in the interest of time. The referee comments are pasted below.

As you will see, the referees acknowledge that the findings are novel and interesting. However, they also have several suggestions for how the study could be strengthened and I think that all points should be addressed. Please let me know if you have any comments or questions regarding the revisions and we can discuss this further, also in a video chat, if you like.

I would thus like to invite you to revise your manuscript with the understanding that the referee concerns must be fully addressed and their suggestions taken on board. Please address all referee concerns in a complete point-by-point response. Acceptance of the manuscript will depend on a positive outcome of a second round of review. It is EMBO reports policy to allow a single round of major revision only and acceptance or rejection of the manuscript will therefore depend on the completeness of your responses included in the next, final version of the manuscript.

We realize that it is difficult to revise to a specific deadline. In the interest of protecting the conceptual advance provided by the work, we recommend a revision within 3 months (9th Jul 2025). Please discuss the revision progress ahead of this time with the editor if you require more time to complete the revisions.

You can either publish the study as a short report or as a full article. For short reports, the revised manuscript should not exceed 27,000 characters (including spaces but excluding materials & methods and references) and 5 main plus 5 expanded view figures. The results and discussion sections must further be combined, which will help to shorten the manuscript text by eliminating some redundancy that is inevitable when discussing the same experiments twice. For a normal article there are no length limitations, but it should have more than 5 main figures and the results and discussion sections must be separate. In both cases, the entire materials and methods must be included in the main manuscript file.

IMPORTANT NOTE: we perform an initial quality control of all revised manuscripts before re-review. Your manuscript will FAIL this control and the handling will be DELAYED if the following APPLIES:
1) A data availability section providing access to data deposited in public databases is missing. If you have not deposited any data, please add a sentence to the data availability section that explains that.
2) Your manuscript contains statistics and error bars based on n=2. Please use scatter blots in these cases. No statistics should be calculated if n=2.

When submitting your revised manuscript, please carefully review the instructions that follow below. Failure to include requested items will delay the evaluation of your revision.

1) a .docx formatted version of the manuscript text (including legends for main figures, EV figures and tables). Please make sure that the changes are highlighted to be clearly visible.

2) individual production quality figure files as .eps, .tif, .jpg (one file per figure). See https://wol-prod-cdn.literatumonline.com/pb-assets/embo-site/EMBOPress_Figure_Guidelines_061115-1561436025777.pdf for more info on how to prepare your figures.

3) We replaced Supplementary Information with Expanded View (EV) Figures and Tables that are collapsible/expandable online. A maximum of 5 EV Figures can be typeset. EV Figures should be cited as 'Figure EV1, Figure EV2" etc... in the text and their respective legends should be included in the main text after the legends of regular figures.

- For the figures that you do NOT wish to display as Expanded View figures, they should be bundled together with their legends in a single PDF file called *Appendix*, which should start with a short Table of Content. Appendix figures should be referred to in the main text as: "Appendix Figure S1, Appendix Figure S2" etc. See detailed instructions regarding expanded view here: <https://www.embopress.org/page/journal/14693178/authorguide#expandedview>

- Additional Tables/Datasets should be labeled and referred to as Table EV1, Dataset EV1, etc. Legends have to be provided in a separate tab in case of .xls files. Alternatively, the legend can be supplied as a separate text file (README) and zipped together with the Table/Dataset file.

4) a .docx formatted letter INCLUDING the reviewers' reports and your detailed point-by-point responses to their comments. As part of the EMBO Press transparent editorial process, the point-by-point response is part of the Review Process File (RPF), which will be published alongside your paper.

5) a complete author checklist, which you can download from our author guidelines <https://www.embopress.org/page/journal/14693178/authorguide>. Please insert information in the checklist that is also

reflected in the manuscript. The completed author checklist will also be part of the RPF.

6) Please note that all corresponding authors are required to supply an ORCID ID for their name upon submission of a revised manuscript (<https://orcid.org/>). Please find instructions on how to link your ORCID ID to your account in our manuscript tracking system in our Author guidelines
<https://www.embopress.org/page/journal/14693178/authorguide#authorshipguidelines>

7) Before submitting your revision, primary datasets produced in this study need to be deposited in an appropriate public database (see https://www.embopress.org/page/journal/14693178/authorguide#datadeposition). Please remember to provide a reviewer password if the datasets are not yet public. The accession numbers and database should be listed in a formal "Data Availability" section placed after Materials & Method (see also
https://www.embopress.org/page/journal/14693178/authorguide#datadeposition). Please note that the Data Availability Section is restricted to new primary data that are part of this study. * Note - All links should resolve to a page where the data can be accessed. *
If your study has not produced novel datasets, please mention this fact in the Data Availability Section.

8) At EMBO Press we ask authors to provide source data for the main manuscript figures. You will receive a separate email with instructions for providing source data with your revised manuscript, including how to upload and organize the files.

9) Our journal also encourages inclusion of *data citations in the reference list* to directly cite datasets that were re-used and obtained from public databases. Data citations in the article text are distinct from normal bibliographical citations and should directly link to the database records from which the data can be accessed. In the main text, data citations are formatted as follows: "Data ref: Smith et al, 2001" or "Data ref: NCBI Sequence Read Archive PRJNA342805, 2017". In the Reference list, data citations must be labeled with "[DATASET]". A data reference must provide the database name, accession number/identifiers and a resolvable link to the landing page from which the data can be accessed at the end of the reference. Further instructions are available at https://www.embopress.org/page/journal/14693178/authorguide#referencesformat

10) Regarding data quantification (see Figure Legends:
https://www.embopress.org/page/journal/14693178/authorguide#figureformat)

The following points must be specified in each figure legend:

- the name of the statistical test used to generate error bars and P values,

- the number (n) of independent experiments (please specify technical or biological replicates) underlying each data point,

- the nature of the bars and error bars (s.d., s.e.m.),

- If the data are obtained from n Program fragment delivered error ``Can't locate object method "less" via package "than" (perhaps you forgot to load "than"?) at //ejpvfs23/sites23b/embor_www/letters/embor_decision_revise_and_review.txt line 56.' 2, use scatter blots showing the individual data points.

Discussion of statistical methodology can be reported in the materials and methods section, but figure legends should contain a basic description of n, P and the test applied.

- Please also include scale bars in all microscopy images.

11) The journal requires a statement specifying whether or not authors have competing interests (defined as all potential or actual interests that could be perceived to influence the presentation or interpretation of an article). In case of competing interests, this must be specified in your disclosure statement. Further information: https://www.embopress.org/competing-interests

12) All Materials and Methods need to be described in the main text using our 'Structured Methods' format, which is required for all research articles. According to this format, the Methods section includes a separate Reagents and Tools Table file (listing key reagents, experimental models, software and relevant equipment and including their sources and relevant identifiers) and a Methods and Protocols section describing the methods using a step-by-step protocol format. The aim is to facilitate adoption of the methodologies across labs. More information on how to adhere to this format as well as a downloadable template (.docx) for the Reagents and Tools Table can be found in our author guidelines:
https://www.embopress.org/page/journal/14693178/authorguide#structuredmethods.

An example of a Method paper with Structured Methods can be found here: https://www.embopress.org/doi/full/10.1038/s44320-024-00037-6#sec-4

We would also welcome the submission of cover suggestions, or motifs to be used by our Graphics Illustrator in designing a

cover.

As part of the EMBO publication's Transparent Editorial Process, EMBO reports publishes online a Review Process File (RPF) to accompany accepted manuscripts. This File will be published in conjunction with your paper and will include the referee reports, your point-by-point response and all pertinent correspondence relating to the manuscript.

You are able to opt out of this by letting the editorial office know (emboreports@embo.org). If you do opt out, the Review Process File link will point to the following statement: "No Review Process File is available with this article, as the authors have chosen not to make the review process public in this case."

I look forward to seeing a revised form of your manuscript when it is ready.

Best wishes,
Esther

Esther Schnapp, PhD
Senior Editor
EMBO reports

Referee #1:

In the manuscript entitled "Modelling human adult V-SVZ niche assembly and ependymal cells generation in brain organoids" Styliani et al. use GEMC1 and MCIDAS overexpression to program human radial glia cells into ependymal cells resulting in the remodeling of niche acquiring features of the adult neurogenic niche in the V-SVZ. The authors set out and employ electroporation of episomal plasmids to deliver genetic information to radial glia cell in human organoids. They find that both, GEMC1 and MCIDAS overexpression results in a reduction in the fraction of proliferative, PAX6 positive radial glia cells and a concomitant increase in P73 positive, FOXJ1 positive, and S100B positive cells. The authors further characterize the cells after GEMC1 and MCIDAS manipulation and conclude that they are multiciliated, often have only short basal process and restructure their immediate surrounding niche forming a hypocellular gap.
The basic question addressed in this manuscript how one of the adult neurogenic niches develops in particular in humans and is timely and highly relevant.
While I see potential in the presented results and hence support eventual publication of this manuscript in EMBO journal, the presented data are premature. I structured my concerns in major and minor points outlined below.

- The characterization of the induced cells is not convincing. That GEMC1-MCIDAS induces a multiciliogenesis program has been shown multiple times before (e.g reviewed in: 10.1016/j.semcdb.2020.04.007). But what is the exact nature of the induced cells, when this program is induced in radial glial cells during development? The authors conclude that the resulting cells are ependymal cells based on the expression of S100B, the other assessed markers (FOXJ1, P73, CEP152, ARL13B) are not specifying a celltype but rather describe multi ciliated cells. However, also choroid plexus cells are multiciliated and express S100B. Moreover, also during fate-specification of Cajal-Retzius cells the MCIDAS-GEMC1 program is used, resulting not in multiciliated cells but in REELIN positive Cajal-Retzius cells 10.1016/j.devcel.2023.05.011 (also a paper which should be cited in the manuscript).
The authors have to characterize the resulting cells in much more detail. Immunohistochemical stainings for choroid plexus (TTR), and Cajal-Retzius cells (REELIN) on the one hand but then also more globally by at least bulk RNAseq. Considering that the very same factors might in fact trigger multiple different cellular programs almost demands a description of the resulting cells on a single cell level, best done by single cell RNAseq. Such a more global approach would also allow to evaluate molecular differences in the resulting cells to the cells found in vivo, differences that like occur given that at the developmental stages used in the study no ependymal cells develop normally as the authors rightly state. Thorough immunohistochemical characterization and quantification might allow to omit this depending on the results.
- The authors mention human-specific characteristics of ependymal cells. What are this human-specific characteristics?
- GEMC1/MCIDAS overexpression results in the formation of hollow structure in the vicinity of the electroporation site. How many times was this found? The information is hard to find. In the figure legend to Figure 4 it say: "Mcidas: b=1, o=6, v=12", the plot in Figure 4G reports roughly 50% showing a hypocellular gap. Does this mean this was seen in 6 ventricles? Please specify this more clearly.
More importantly, is there a correlation with the number of electroporated cells, i.e. is there a lower limit of electroporated cells necessary to induce the formation of a hypocellular gap?
- How would ependymal cells have such a cell-non-autonomous effect on tissue architecture? Again, more global transcriptomic data would help tremendously to formulate hypotheses on this fundamental question raised by the findings in the manuscript. A discussion on this aspect should be integrated in the manuscript.
- The centriole amplification in Figure 3M-O is hardly convincing. Please provide better quality pictures including other markers such as gamma-tubulin for the gamma-TuRC, and Pericentrin.
- Ependymal cells have cobblestone like organization forming pinwheel structures in the V-SVZ. The authors should describe

the cellular morphology of the resulting cells including high resolution en-face pictures to elucidate whether such cell and tissue architectonic aspects crucial for a functional adult neurogenic niche are induced by MCIDAS-GEMC1

Minor:
- In the abstract the abbreviation aRG is used, however at that position in the manuscript the abbreviation is not introduced yet.
- There is no quantification of the extent of overexpression by electroporation of MCIDAS and GEMC1. qPCR of FACS sorted cells could be one way to do that.
- The authors refer to ventricular-like areas. This should rather be something like ventricular-zone like areas.
- The authors refer to neural somata (cell bodies stained with NEUN). the authors are referering to neuronal somata. Neural somata would be all neural cells, including radial glia, ependyma, choroid plexus etc, neuronal only the soma of neurons.
- Consistency: sometimes GemC1 mostly GEMC1, sometimes Mcidas mostly MCIDAS, in particular in the figure legends.

Referee #2:

In their manuscript, the authors exploit organoid technology to model the human V-SVZ niche and focus on the generation of ependymal cells. In most organoid models, ependymal cells are not present in significant amounts. Thus the study, especially of human, ependymal cell biology and how they contribute to the adult V-SVZ is not easily feasible. To approach this problem, the authors have overexpressed two genes (GEMC1 and MCIDAS, essential for endogenous V-SVZ assembly) via electroporation in organoids. In their experimental paradigms they could 'reprogram' radial glial cells so that they precociously differentiated into ependymal cells at high frequency. Consequently, the ependymal cells then conferred characteristics of the V-SVZ in human organoid cultures.

The authors have addressed an important aspect in human organoid biology/technology. The experiments have been carried out mostly well and the manuscript is very clearly written. Some aspects of the presentation could be improved to enhance the reading experience.

1. In the introduction, the authors should explain what GEMC1 and MCIDAS are encoding for and introduce a bit more the function of the respective proteins and pathways. Such information and some more background on these pathways will help the broader readership to understand the context better.
2. At many instances throughout the manuscript the authors state '...identify the molecular mechanisms that regulate human ependymal cell generation...'. In my opinion these claims go beyond what is actually presented in the manuscript. They use overexpression paradigms but do not look into the molecular mechanisms in general and also not more specifically how GEMC1 and MCIDAS regulate cell fate decision. The authors should thus tone down these statements.
3. Page 7, top line. The authors mention '...not highly expressed in the human cortex...' but they look at data from organoids. They should not use human brain/cortex if referring to organoid data. They should carefully rephrase respective sentences actually throughout the manuscript, also in the subsequent and especially Discussion section (e.g. page 13, last line and subsequent paragraphs).
4. The authors generate ependymal cells precociously. Are these cells 'normal' or should one expect differences due to the overexpression of GEMC1/MCIDAS. While the authors may not have experimental data, they should at least discuss in some more detail in how far differences to the normal state (i.e. endogenous human ependymal cells versus organoid ependymal cells, that overexpress GEMC1/MCIDAS) could occur.
5. In Figure 4, the authors document the hypocellular gap but the images are quite dim, especially the D',E' and F' panels. Could the authors utilize another cell marker and/or increase the gain while imaging the DAPI signal so that the cellular architecture becomes visible better.

We would like to thank the editor and reviewers for their valuable feedback and suggestions, which we believe have greatly enhanced the quality of our manuscript.

In response to the reviewers' comments, we conducted additional experiments to:

- characterize further the cellular identity of the *GEMC1* and *MCIDAS* electroporated cells, using markers specific for alternative cell types that employ the GEMC1/MCIDAS pathway during their development. Specifically, we used REELIN for Cajal-Retzius neurons (Figure 2D-F''''), TTR for choroid plexus cells (Figure 2G-I''), OLIG2 for oligodendrocytes (Figure EV2G-I''), GFAP for astrocytes and adult neural stem cells (Figure 5I-K''') and SOX2 (Figure EV5A-C''') for neural stem cells.

- further highlight the centriole amplification occurring in the *GEMC1* and *MCIDAS* electroporated cells, using the centriolar markers γ-TUBULIN and CEP-164 (Figure 4D-I'').

- further characterize GEMC1's and MCIDA's ability to influence the V-SVZ niche cytoarchitecture in the electroporated brain organoids, by en-face imaging of thick organoid sections using the markers GFAP and SOX2 (Figure 5I-K''', Figure EV5A-C''').

In addition, modifications were made to the phrasing and structuring of the manuscript in order to adhere to the word limits set by the journal (short report format – limited total characters must be kept).

All changes in the text that were made in response to reviewers' comments are highlighted in yellow in the manuscript and a point-by-point response to reviewers' comments can be found below.

Referee #1:

In the manuscript entitled "Modelling human adult V-SVZ niche assembly and ependymal cells generation in brain organoids" Styliani et al. use GEMC1 and MCIDAS overexpression to program human radial glia cells into ependymal cells resulting in the remodeling of niche acquiring features of the adult neurogenic niche in the V-SVZ. The authors set out and employ electroporation of episomal plasmids to deliver genetic information to radial glia cell in human organoids. They find that both, GEMC1 and MCIDAS overexpression results in a reduction in the fraction of proliferative, PAX6 positive radial glia cells and a concomitant increase in P73 positive, FOXJ1 positive, and S100B positive cells. The authors further characterize the cells after GEMC1 and MCIDAS manipulation and conclude that they are multiciliated, often have only short basal process and restructure their immediate surrounding niche forming a hypocellular gap.

The basic question addressed in this manuscript how one of the adult neurogenic niches develops in particular in humans and is timely and highly relevant.

While I see potential in the presented results and hence support eventual publication of this manuscript in EMBO journal, the presented data are premature. I structured my concerns in major and minor points outlined below.

Response to Referee #1 comments:

We would like to thank Referee #1 for taking the time to read and evaluate our manuscript. Their comments were well-thought out and helped us better understand the steps necessary to strengthen the integrity of our data. Please find our responses to the points raised below.

Major comments

- The characterization of the induced cells is not convincing. That GEMC1-MCIDAS induces a multiciliogenesis program has been shown multiple times before (e.g reviewed in: 10.1016/j.semcdb.2020.04.007). But what is the exact nature of the induced cells, when this program is induced in radial glial cells during development? The authors conclude that the resulting cells are ependymal cells based on the expression of S100B, the other assessed markers (FOXJ1, P73, CEP152, ARL13B) are not specifying a celltype but rather describe multi ciliated cells. However, also choroid plexus cells are

multiciliated and express S100B. Moreover, also during fate-specification of Cajal-Retzius cells the MCIDAS-GEMC1 program is used, resulting not in multiciliated cells but in REELIN positive Cajal-Retzius cells 10.1016/j.devcel.2023.05.011 (also a paper which should be cited in the manuscript).

The authors have to characterize the resulting cells in much more detail. Immunohistochemical stainings for choroid plexus (TTR), and Cajal-Retzius cells (REELIN) on the one hand but then also more globally by at least bulk RNAseq. Considering that the very same factors might in fact trigger multiple different cellular programs almost demands a description of the resulting cells on a single cell level, best done by single cell RNAseq. Such a more global approach would also allow to evaluate molecular differences in the resulting cells to the cells found in vivo, differences that like occur given that at the developmental stages used in the study no ependymal cells develop normally as the authors rightly state. Thorough immunohistochemical characterization and quantification might allow to omit this depending on the results.

We would like to thank the reviewer for these valid and well-thought-out observations. Indeed, the GEMC1/MCIDAS pathway has been shown to be active in brain cell types other than ependymal cells, namely in the cells found in the choroid plexus and transiently in the developing Cajal-Retzius neurons.

As the reviewer aptly suggested, RNA sequencing would be greatly beneficial in characterizing the cell identity of the GEMC1 and MCIDAS overexpressing cells. Towards this end, we generated human brain organoids and electroporated them with plasmids encoding GFP, GEMC1-GFP or MCIDAS-GFP at 30-days old and sorted GFP expressing cells 10 days after the electroporation. Aiming for a large-scale experiment, we utilized organoids from three different batches and lysed more than 20 organoids per sample, having three biological replicates per condition. Before lysing them, we observed the organoids in a fluorescent stereoscope to ensure that their electroporation was successful. We sorted the GFP+ cells in a BD Aria III FACS machine and proceeded to isolate their RNA (Figure only for reviewers).

As mentioned in the manuscript, GEMC1 and MCIDAS electroporated cells exit the cell cycle, so they do not proliferate and thus there is no internal expansion of their cell numbers. Additionally, through the experimental setup employed here, namely acute and targeted electroporation in the cells adjacent to the ventricle-like structures of the organoids, only a limited number of ventricles can be subjected to electroporation, as we can only manipulate the ventricles that are on the outer surface of the organoids, and can be visible through a stereoscope (Rapti and Kyrousi, 2025). For these reasons, the number of the sorted GFP+ cells was very low, and subsequently the

quantity and quality of the isolated RNA was deemed insufficient for bulk RNA sequencing, which was similar to all conditions and batches. Thus, we could not apply this strategy for the characterization of the electroporated cells when GMEC1 or MCIDAS is overexpressed in human neural progenitors in brain organoids (Figure only for reviewers).

To examine whether GEMC1 or MCIDAS ectopic expression in radial glial cells may lead to their differentiation towards cell types other than ependyma, we implemented an alternative method, following the reviewer's suggestion, and performed immunofluorescence stainings in brain organoids 10-days post electroporation. We used the marker REELIN, which is specific for Cajal-Retzius neurons and the marker TTR, which is specific for choroid plexus cells. In our experimental setup, we did not observe any GFP+ electroporated cells that co-expressed REELIN or TTR, indicating that the manipulated radial glial cells did not differentiate into Cajal-Retzius or choroid plexus cells (Figure 2D-I").

Additionally, we performed immunofluorescence stainings at organoids 21-days post electroporation using the marker OLIG2 to examine whether GEMC1 and MCIDAS electroporated cells express markers associated with oligodendrocytes. As before, we did not observe any GFP+ electroporated cells expressing this marker, indicating that the manipulated cells did not acquire an oligodendrocyte identity (Figure EV2G-I").

Lastly, we performed immunofluorescence experiments using the neural stem cell marker SOX2 and GFAP for astroglial-like cells (astrocytes or adult neural stem cells). As expected, we observed some SOX2+GFP+ cells in the GEMC1 and MCIDAS manipulated organoids (Figure EV5A-C'"). These cells represent electroporated apical radial glial cells that did not experience a cell fate switch. This comes in accordance with our observations on the immunostaining we performed with the apical radial glial marker PAX6, where we also observed some PAX6+GFP+ cells (Fig. 1K-M", Fig. EV1H-J"). On the contrary, we did not observe any GFAP+GFP+ adult neural stem cells or astrocytes (Figure 5I-K'").

In order to examine the cell-non-autonomous effect of ependymal cells on tissue architecture we performed en face imaging of GEMC1 and MCIDAS electroporated organoids 10-days post their electroporation, investigating the formation of GFP+ pinwheel-like structures. For this analysis we performed stainings using the markers GFAP and SOX2, alongside Phalloidin. We did observe GFP+ pinwheel-like structures (see response in Reviewers' 1 comment 6), and also some SOX2+GFP+ cells, outside of the neural rosettes. These cells could be indicative of incomplete ependymal programming or could represent a transient progenitor of neural stem cells and ependymal cells. Iin mice, it has been shown that these two cell types are generated

through a common glial lineage (Fuentealba et al., 2015; Merkle et al., 2004; Spassky et al., 2005). Whether they share a common ancestor in humans needs to be investigated further, however this is out of the scope of the present work.

In support of our results, previous studies using the same plasmids, have shown that GEMC1 or MCIDAS ectopic expression in cultured primary mouse radial glial cellsand astrocytes, as well as in mouse embryonic stem cells, leads to their differentiation towards ependymal cells only and not in choroid plexus or Cajal-Retzius cells. In addition, in vivo in utero electroporation experiments in mice using the same constructs have led to the differentiation of periventricular cells (aRGs) in ependymal cells and not in other cell types (Kyrousi et al., 2015; Kaplani et al., 2024). So, the ependymal-specific differentiation of GEMC1/MCIDAS electroporated cells has been well established in the murine model. In our manuscript we propose that the same principle applies to overexpressing human radial glial cells.

Figure for referee with unpublished data and its description has been removed upon request by the authors.

(Page 4, lines 61-63): "In addition, GEMC1/MCIDAS pathway…… the Cajal-Retzius neurons."

And

(Page 8, lines 140-148): "Given that the GEMC1/MCIDAS pathway is activated during…… in the GEMC1 and MCIDAS OE BOs."

And

(Page 11, lines 225-231): "Aiming to investigate whether this phenotype…. role of ECs on normal hV-SVZ niche assembly."

- The authors mention human-specific characteristics of ependymal cells. What are these human-specific characteristics?

*The human-specific characteristic of ependymal cells that we mention in our manuscript is the basal process that extends perpendicularly away from the ventricles and towards the hypocellular gap (*Quiñones-Hinojosa et al., 2006)*.*

This information has been made clearer in the text of the manuscript, as suggested by the reviewer, and can be found in:

(Page 3, lines 41-42): "….extend a basal process perpendicularly to Layer II. This short basal process distinguishes hECs from those of mice."

- GEMC1/MCIDAS overexpression results in the formation of hollow structure in the vicinity of the electroporation site. How many times was this found? The information is hard to find. In the figure legend to Figure 4 it say: "Mcidas: b=1, o=6, v=12", the plot in Figure 4G reports roughly 50% showing a hypocellular gap. Does this mean this was seen in 6 ventricles? Please specify this more clearly.

More importantly, is there a correlation with the number of electroporated cells, i.e. is there a lower limit of electroporated cells necessary to induce the formation of a hypocellular gap?

We thank the referee for pinpointing this data misinterpretation gap. Indeed the 50% of hypocellular gap presenting ventricles corresponds to a number of 6 ventricles in the MCIDAS electroporated organoids and 12 ventricles in the GEMC1 electroporated organoids. This information was made clearer in the manuscript and can be found as follows:

(Page 11, lines 216-219): "In the manipulated BOs this hypocellular gap…. not observed in the control condition (Fig. 5G)."

In addition, we have analyzed whether the number of GEMC1 or MCIDAS electroporated cells correlates with the formation of a hypocellular gap, and we concluded that indeed it does. More specifically, in the GEMC1 or MCIDAS electroporated ventricles that present a hypocellular gap, the number of the electroporated cells is almost double that of the GEMC1 or MCIDAS electroporated cells in the ventricles where there is no gap formation. We have set a threshold of 50 GEMC1 or MCIDAS electroporated cells, above which we observe hypocellular gap formation (Figure 5H). It is worth noting that the GFP only electroporated cells surpass this set threshold, thus no gap is observed as expected (control condition).

This new data can be found in the manuscript and new figures as follows:

(Page 11, lines 219-222): "Moreover, there is positive correlation… a hypocellular gap is observed (Fig. 5H)."

- How would ependymal cells have such a cell-non-autonomous effect on tissue architecture? Again, more global transcriptomic data would help tremendously to formulate hypotheses on this fundamental question raised by the findings in the manuscript. A discussion on this aspect should be integrated in the manuscript.

We thank the reviewer for this important inquiry. Indeed, transcriptomic data derived from GEMC1 or MCIDAS electroporated organoids would be greatly beneficial for addressing this question. Though we tried to approach this matter through a bulk RNAseq analysis, it was deemed unsuccessful due to

the nature of our experimental setup (see response in Reviewers' 1 comment 1 and Figure only for reviewers). This was discussed more extensively in our response to the first major comment.

Nevertheless, previous studies have shown that ependymal cells play a crucial role in the proper formation of the V-SVZ niche. Studies have shown that ependymal cells are responsible for the enlargement of brain ventricles during development, through the enlargement of their apical surface, while their function is crucial for the proper assembly of the V-SVZ niche in mice (Quaresima et al., 2022; Redmond et al., 2019b). They also help shape the region structurally through the formation of characteristic pinwheel structures alongside adult neural stem cells (*Mirzadeh et al., 2008). These pinwheel structures have also been identified in human tissues, highlighting the role of ependymal cells on human V-SVZ niche assembly (Coletti et al., 2018). Towards verifying this in our experimental set up we performed en face imaging of GEMC1 and MCIDAS electroporated brain organoids, and we observed the formation of GFP+ pinwheel-like structures (see response in Reviewers' 1 comment 6).*

This information has been added to the manuscript, and can be found in:

(Page 4, lines 67-71): "The cell-non-autonomous role of the EC differentiation…. and impaired adult neurogenesis."

Besides, we include discussion on this in the revised manuscript:

(Page 11, lines 225-231): "Aiming to investigate…..on normal hV-SVZ niche assembly."

- The centriole amplification in Figure 3M-O is hardly convincing. Please provide better quality pictures including other markers such as gamma-tubulin for the gamma-TuRC, and Pericentrin.

We thank the reviewer for this suggestion. We performed additional immunostainings using the markers γ-TUBULIN and CEP-164, to further highlight the accumulation of basal bodies on the GEMC1 or MCIDAS electroporated cells (Figure 4D-I").

These new data can be found in the manuscript in:

(Page 9, lines 179-181): "At the GEMC1 and MCIDAS OE BOs…. associated with mature ECs."

- Ependymal cells have cobblestone like organization forming pinwheel structures in the V-SVZ. The authors should describe the cellular morphology

of the resulting cells including high resolution en-face pictures to elucidate whether such cell and tissue architectonic aspects crucial for a functional adult neurogenic niche are induced by MCIDAS-GEMC1

We would like to thank the reviewer for this important suggestion. Indeed, it has been shown that ependymal cells alongside adult neural stem cells form characteristic pinwheel structures at the surface of the V-SVZ niche, both in the murine model and in humans (Coletti et al., 2018; Mirzadeh et al., 2008).

Brain organoids' ventricular-like areas do not have a specific orientation or position within the tissue, like the murine brain does, which results in the generation of ventricular-like structures in random places within the brain organoids, so en-face imaging cannot be performed following a specific protocol, due to difficulties in dissecting the areas properly. In order to address this inquiry, we have sectioned electroporated brain organoids in thick sections of 30μm in a cryotome to preserve as much as possible the 3D organization of the electroporated area, and performed immunostainings using the markers GFAP or SOX2 along with Phalloidin (Figure 5I-K''', Figure EV5A-C'''). Through imaging analysis, we identified electroporated areas that resembled the expected en-face cellular organization of a V-SVZ area (clusters of GFP+ cells in close proximity to one another). Then, we critically searched for GFAP+ or SOX2+ cells that were surrounded by GFP+ cells with visible Phalloidin-stained borders that resembled a pinwheel, and observed the formation of GFP+ pinwheel-like structures at the GEMC1 and MCIDAS electroporated brain organoids. This further supports our suggestion on the cell-non-autonomous role of ependymal cells on normal human V-SVZ niche assembly, which we also discuss in the revised manuscript.

These new data have been included in the manuscript in:

(Page 11, lines 225-231): "Aiming to investigate…..on normal hV-SVZ niche assembly."

Minor comments

- In the abstract the abbreviation aRG is used, however at that position in the manuscript the abbreviation is not introduced yet.

We thank the reviewer for pinpointing this. The abbreviation has been replaced with the full name of the cell type mentioned. We have also checked the whole manuscript for more typos and made the appropriate changes accordingly.

(Page 2, line 23): "….apical radial glial cells…."

- There is no quantification of the extent of overexpression by electroporation of MCIDAS and GEMC1. qPCR of FACS sorted cells could be one way to do that.

We thank the reviewer for highlighting this information gap. We planned to answer this question through qPCR analysis of FACS sorted cells as suggested, but the RNA isolation of the electroporated cells was unsuccessful due to their limited numbers (see response in Reviewers' 1 comment 1 and Figure only for reviewers). This has been analyzed more extensively in our response to the first major comment.

In order to quantify the overexpression by electroporation of MCIDAS we have performed an immunostaining of electroporated organoids using an antibody against MCIDAS (Figure EV3C-C"). We have quantified the expression of the marker and showed that 71.57% of the MCIDAS electroporated cells express MCIDAS (Figure EV3M).

As established in prior research, GEMC1 is an upstream activator of MCIDAS (Arbi et al., 2016; Terré et al., 2016). So, as there is no antibody available for the detection of GEMC1, we also performed immunostaining of GEMC1 electroporated organoids using MCIDAS as a marker (Figure EV3B-B"). We have quantified the results and showed that 53.31% of the GEMC1 electroporated cells express MCIDAS, while in the control there were no GFP+MCIDAS+ cells detected (Figure EV3M). Even though the expression of MCIDAS is not a direct reflection of the expression of GEMC1, as we cannot claim that all GEMC1-expressing cells will successfully express MCIDAS, we can state that at least 53.51% of them do. This is also supported by the fact that in our control organoids there is no expression of endogenous MCIDAS at this stage (Figure EV3A-A", Figure EV3M), so the MCIDAS expression in the GEMC1 electroporated cells is directly linked to the expression of GEMC1.

Moreover, the constructs used in our experiments have been used multiple times in previous studies, where their overexpression capacity and effect (Kaplani et al., 2025; Lalioti et al., 2019b; Ortiz-Álvarez et al., 2019; Kyrousi et al., 2015) have been well-documented. Regarding the overexpression capacity of the plasmids, in situ hybridization experiments performed on brain sections of mice after in utero electroporation, clearly show the robust expression of GEMC1 and MCIDAS in the experimental conditions, in contrast to the control (Figure only for reviewers). Taking into account all these data, we are confident that the GEMC1 and MCIDAS plasmids we employed in the experiments presented in this manuscript show a robust expression.

This information has been stated more clearly in the manuscript and can be found in:

(Page 9, lines 162-164): "In mice, it was shown that GEMC1…. at 4dpe (Fig. EV3A-C", M)."

- The authors refer to ventricular-like areas. This should rather be something like ventricular-zone like areas.

The wording has been changed throughout the manuscript, as the reviewer suggested.

- The authors refer to neural somata (cell bodies stained with NEUN). the authors are referering to neuronal somata. Neural somata would be all neural cells, including radial glia, ependyma, choroid plexus etc, neuronal only the soma of neurons.

We thank the reviewer for pointing out this miswording. The wording has been corrected in the manuscript.

(Page 8, line 139): "…neuronal somata…"

- Consistency: sometimes GemC1 mostly GEMC1, sometimes Mcidas mostly MCIDAS, in particular in the figure legends.

We thank the reviewer for pointing out this inconsistency. All gene names have been changed to capital lettering throughout the manuscript.

Referee #2:

In their manuscript, the authors exploit organoid technology to model the human V-SVZ niche and focus on the generation of ependymal cells. In most organoid models, ependymal cells are not present in significant amounts. Thus the study, especially of human, ependymal cell biology and how they contribute to the adult V-SVZ is not easily feasible. To approach this problem, the authors have overexpressed two genes (GEMC1 and MCIDAS, essential for endogenous V-SVZ assembly) via electroporation in organoids. In their experimental paradigms they could 'reprogram' radial glial cells so that they precociously differentiated into ependymal cells at high frequency. Consequently, the ependymal cells then conferred characteristics of the V-SVZ in human organoid cultures.

The authors have addressed an important aspect in human organoid biology/technology. The experiments have been carried out mostly well and the manuscript is very clearly written. Some aspects of the presentation could be improved to enhance the reading experience.

Response to Referee #2 comments:

We would like to thank Referee #2 for taking the time to read and evaluate our manuscript. Their comments were invaluable in improving our manuscript and presenting our data in a clearer and scientifically correct manner. Please find our responses to the points raised below.

1. In the introduction, the authors should explain what GEMC1 and MCIDAS are encoding for and introduce a bit more the function of the respective proteins and pathways. Such information and some more background on these pathways will help the broader readership to understand the context better.

We thank the referee for pinpointing this literature gap. More information regarding the functions and pathways of GEMC1 and MCIDAS has been added to the manuscript and can be found in:

(Page 4, lines 56-63): "GEMC1 and MCIDAS have been identified….membrane docking of ECs' basal bodies."

2. At many instances throughout the manuscript the authors state '...identify the molecular mechanisms that regulate human ependymal cell generation...'. In my opinion these claims go beyond what is actually presented in the manuscript. They use overexpression paradigms but do not look into the molecular mechanisms in general and also not more specifically how GEMC1 and MCIDAS regulate cell fate decision. The authors should thus tone down these statements.

We thank the referee for pointing this out. Indeed, a misinterpretation of our presented data could occur because of our previous phrasing. We changed our wording throughout the manuscript, to more precisely portray the information derived from our overexpression experiments.

This information has been made clearer in the text of the manuscript, as suggested by the reviewer, and can be found throughout the revised manuscript – some examples are reported also here as follows:

(Page 4, lines 78-79): "Toward deciphering haV-SVZ niche assembly…. the formation of the niche."

And

(Page 12, lines 253-256): "Summarizing, we propose….. acquiring haV-SVZ niche characteristics….)

3. Page 7, top line. The authors mention '...not highly expressed in the human cortex...' but they look at data from organoids. They should not use human brain/cortex if referring to organoid data. They should carefully rephrase respective sentences actually throughout the manuscript, also in the subsequent and especially Discussion section (e.g. page 13, last line and subsequent paragraphs).

We thank the referee for pointing this out. The wording has been changed throughout the manuscript, so as to avoid misinterpretation of our presented data.

4. The authors generate ependymal cells precociously. Are these cells 'normal' or should one expect differences due to the overexpression of GEMC1/MCIDAS. While the authors may not have experimental data, they should at least discuss in some more detail in how far differences to the normal state (i.e. endogenous human ependymal cells versus organoid ependymal cells, that overexpress GEMC1/MCIDAS) could occur.

An addition to the discussion has been made in order to address the reviewer's comment.

The new information can be found in:

(Page 10, lines 192-195): "In our human-specific model…. and endogenous hECs."

5. In Figure 4, the authors document the hypocellular gap but the images are quite dim, especially the D',E' and F' panels. Could the authors utilize another cell marker and/or increase the gain while imaging the DAPI signal so that the cellular architecture becomes visible better.

We thank the reviewer for pointing this out. The images in question have been modified so that the cellular architecture described becomes more visible. The new images can be found in Figure 5D-F".

Dear Prof. Kyrousi,

Thank you for the submission of your revised manuscript. We have now received the enclosed report from referee 1 who was asked to assess it, and I am happy to say that this referee supports the publication of your study now. Only a few editorial requests will need to be addressed before we can proceed with the official acceptance of your manuscript:

- Please rename the "Data and code availability" subheading to "Data Availability Section".

- The ms author list needs to have the same format as in our online submission system: First Name Last Name; name discrepancy Giaimo in the ms vs. Gaimo in the system; emails of all 3 corr. authors are needed on the ms title page.

- In the REFERENCE list, DOIs should only be used for preprints and datasets that have not been published yet; Bibliography needs to be updated to References.

- FIGURE CALLOUTS are missing for Fig. 5DEF, please add.

- The Reagents & Tools TABLE needs to be removed from the ms file and uploaded separately.

- Materials and Methods should be Methods.

- Our routine image analysis identified a possible reuse in Figure EV3 1st and 3rd lane that is Not listed in the figure legend. Can you please clarify.

Figure Legends - Comments

- Please note that the exact p values are not provided in the legends of figures 1N, O; 3J, K, L; 5G, EV3 M-P, please provide exact values as reasonable.

I would like to suggest some minor changes to the title and abstract that needs to be written in present tense. Do you agree with this:

Modelling human adult V-SVZ niche assembly and ependymal cell generation in brain organoids

The V-SVZ niche is vital for adult neurogenesis in mammals, yet its regulation in humans remains poorly understood. Current models, including brain organoids, fail to replicate the unique cytoarchitecture of this niche, particularly the multiciliated ependymal cells, which are essential for its function and organization. Here, we utilize GEMC1 and MCIDAS to program human apical radial glial cells into ependymal cells, employing human brain organoids as a model. This approach induces premature ependymal cell differentiation and reorganization of the embryonic neurogenic niche, conferring characteristics of the human adult V-SVZ niche. Our findings highlight a molecular pathway that leads to ependymal cell generation and adult human V-SVZ niche reconstruction, providing a platform to study its development and function.

I also modified the summary and bullet points. Are you OK with this:

Human progenitors are programmed into ependymal cells in brain organoids via GEMC1 and MCIDAS expression. This strategy recapitulates hallmarks of the adult human V-SVZ niche, providing a platform to investigate its regulation and function.
• GEMC1 and MCIDAS induce human ependymal cell generation
• Programmed ependymal cells exhibit intrinsic human-specific characteristics
• Ependymal cell generation promotes human adult V-SVZ niche assembly

The synopsis image looks good but I am not sure that it can be 800 pixels high. I will let you know in case we need a slightly smaller image.

I look forward to seeing a final version of your manuscript as soon as possible.

Best regards,

Esther

Esther Schnapp, PhD
Senior Editor
EMBO reports

Referee #1:

In the revised version of the manuscript entitled "Modelling human adult V-SVZ niche assembly and ependymal cells generation in brain organoids" the authors Styliani et al. addressed a considerable fraction of the reservations I had towards the manuscript in the first submission. In particular the more thorough characterization of the celltypes produced after manipulation of GEMC1 and MCIDAS. It is unfortunate that the unbiased molecular characterization of the effects induced by GEMC1 and MCIDAS overexpression by bulk RNAseq did not work. However, I do appreciate the difficulties to extract electroporated cells from organoids and extract high-quality RNA for library preparation.
Taken together, the increased quality in the characterization of the electroporated cells, the adaptation in the manuscript and the important basic question how the adult neurogenic niches develop in humans, I support publication of this manuscript in it current form.

We would like to thank the editor for their valuable suggestions, which we believe have greatly polished our manuscript.

In response to the editor’s comments, we modified the phrasing and structuring of the manuscript.

All changes in the text that were made in response to the editor’s comments are highlighted in green in the manuscript and a point-by-point response to the editor’s suggestions can be found below.

Editor's suggestions

1. Please rename the "Data and code availability" subheading to "Data Availability Section".

The name of this section has been changed.

2. The ms author list needs to have the same format as in our online submission system: First Name Last Name; name discrepancy Giaimo in the ms vs. Gaimo in the system; emails of all 3 corr. authors are needed on the ms title page.

The correct name of the author (Giaimo) will be used in the online submission system. The emails of the corresponding authors have been added to the title page.

3. In the REFERENCE list, DOIs should only be used for preprints and datasets that have not been published yet; Bibliography needs to be updated to References.

The name of this section has been changed and the DOIs have been deleted as necessary.

4. FIGURE CALLOUTS are missing for Fig. 5DEF, please add.

The figure callouts have been added to the text and can be found in page 12, line 228.

5. The Reagents & Tools TABLE needs to be removed from the ms file and uploaded separately.

The table has been removed from the manuscript and will be uploaded as a separate file.

6. Materials and Methods should be Methods.

The name of this section has been changed.

7. Our routine image analysis identified a possible reuse in Figure EV3 1st and 3rd lane that is Not listed in the figure legend. Can you please clarify.

You identified an image reuse in Figure EV3, specifically in the 1st and 3rd lanes. Indeed, the confocal images (raw data) of the panels A-A" and B-B" are the same confocal images that were used for the panels G-G" and H-H", respectively. In both cases, this occurs because we performed a co-staining on the organoid slices using antibodies against GFP, MCIDAS and FOXJ1 together. As in each lane we wanted to emphasise a specific cellular marker (1st lane: MCIDAS, 3rd lane: FOXJ1), we used the same original images (thus channels for DAPI and GFP are the same) but presented the appropriate marker in each lane. We decided to arrange the figure like that

because the important information is not the triple-positive (GFP+MCDAS+FOXJ1+) cells. This is also true for the panels F-F" and I-I", where the same raw image was used, because of co-staining with antibodies against GFP, P73 and FOXJ1 were used simultaneously. We hope that you agree with us. In case you want us to change the figures, we will follow your suggestion.

8. Please note that the exact p values are not provided in the legends of figures 1N, O; 3J, K, L; 5G, EV3 M-P, please provide exact values as reasonable.

The p values of the abovementioned graphs have been added to the corresponding figure legends.

9. I would like to suggest some minor changes to the title and abstract that needs to be written in present tense. Do you agree with this:

Modelling human adult V-SVZ niche assembly and ependymal cell generation in brain organoids.

The V-SVZ niche is vital for adult neurogenesis in mammals, yet its regulation in humans remains poorly understood. Current models, including brain organoids, fail to replicate the unique cytoarchitecture of this niche, particularly the multiciliated ependymal cells, which are essential for its function and organization. Here, we utilize GEMC1 and MCIDAS to program human apical radial glial cells into ependymal cells, employing human brain organoids as a model. This approach induces premature ependymal cell differentiation and reorganization of the embryonic neurogenic niche, conferring characteristics of the human adult V-SVZ niche. Our findings highlight a molecular pathway that leads to ependymal cell generation and adult human V-SVZ niche reconstruction, providing a platform to study its development and function.

We do agree with the editor's suggestions and have changed the title and abstract of the manuscript to present tense.

10. I also modified the summary and bullet points. Are you OK with this:

Human progenitors are programmed into ependymal cells in brain organoids via GEMC1 and MCIDAS expression. This strategy recapitulates hallmarks of the adult human V-SVZ niche, providing a platform to investigate its regulation and function.

- GEMC1 and MCIDAS induce human ependymal cell generation
- Programmed ependymal cells exhibit intrinsic human-specific characteristics
- Ependymal cell generation promotes human adult V-SVZ niche assembly

We do agree with the changes and have implemented them in our summary and bullet points.

2nd Revision - Editorial Decision 23rd Oct 2025

Prof. Christina Kyrousi
National and Kapodistrian University of Athens, Greece
First Department of Psychiatry, Medical School
Greece

Dear Christina,

I am very pleased to accept your manuscript for publication in the next available issue of EMBO reports. Thank you for your contribution to our journal.

Your manuscript will be processed for publication by EMBO Press. It will be copy edited and you will receive page proofs prior to publication. Please note that you will be contacted by Springer Nature Author Services to complete licensing and payment information.

You may qualify for financial assistance for your publication charges - either via a Springer Nature fully open access agreement or an EMBO initiative. Check your eligibility: https://www.embopress.org/page/journal/14693178/authorguide#chargesguide

Should you be planning a Press Release on your article, please get in contact with embo_production@springernature.com as early as possible in order to coordinate publication and release dates.

If you have any questions, please do not hesitate to contact the Editorial Office. Thank you for your contribution to EMBO Reports.

Best regards,
Esther

Esther Schnapp, PhD
Senior Editor
EMBO reports

>>> Please note that it is EMBO Reports policy for the transcript of the editorial process (containing referee reports and your response letter) to be published as an online supplement to each paper. If you do NOT want this, you will need to inform the Editorial Office via email immediately. More information is available here: https://www.embopress.org/transparent-process#Review_Process

EMBO Press Author Checklist

Corresponding Author Name: Kyrousi Christina, Cappello Silvia, Taraviras Stavros
Journal Submitted to: EMBO Reports
Manuscript Number: EMBOR-2025-61355V1

USEFUL LINKS FOR COMPLETING THIS FORM
The EMBO Journal - Author Guidelines
EMBO Reports - Author Guidelines
Molecular Systems Biology - Author Guidelines
EMBO Molecular Medicine - Author Guidelines

Reporting Checklist for Life Science Articles (updated January
This checklist is adapted from Materials Design Analysis Reporting (MDAR) Checklist for Authors. MDAR establishes a minimum set of requirements in transparent reporting in the life sciences (see Statement of Task: 10.31222/osf.io/9sm4x). Please follow the journal's guidelines in preparing your
Please note that a copy of this checklist will be published alongside your article.

Abridged guidelines for figures
1. Data
The data shown in figures should satisfy the following conditions:
- the data were obtained and processed according to the field's best practice and are presented to reflect the results of the experiments in an accurate and unbiased manner.
- ideally, figure panels should include only measurements that are directly comparable to each other and obtained with the same assay.
- plots include clearly labeled error bars for independent experiments and sample sizes. Unless justified, error bars should not be shown for technical
- if n<5, the individual data points from each experiment should be plotted. Any statistical test employed should be justified.
- Source Data should be included to report the data underlying figures according to the guidelines set out in the authorship guidelines on Data

2. Captions
Each figure caption should contain the following information, for each panel where they are relevant:
- a specification of the experimental system investigated (eg cell line, species name).
- the assay(s) and method(s) used to carry out the reported observations and measurements.
- an explicit mention of the biological and chemical entity(ies) that are being measured.
- an explicit mention of the biological and chemical entity(ies) that are altered/varied/perturbed in a controlled manner.
- the exact sample size (n) for each experimental group/condition, given as a number, not a range;
- a description of the sample collection allowing the reader to understand whether the samples represent technical or biological replicates (including how many animals, litters, cultures, etc.).
- a statement of how many times the experiment shown was independently replicated in the laboratory.
- definitions of statistical methods and measures:
  - common tests, such as t-test (please specify whether paired vs. unpaired), simple χ2 tests, Wilcoxon and Mann-Whitney tests, can be unambiguously identified by name only, but more complex techniques should be described in the methods section;
  - are tests one-sided or two-sided?
  - are there adjustments for multiple comparisons?
  - exact statistical test results, e.g., P values = x but not P values < x;
  - definition of 'center values' as median or average;
  - definition of error bars as s.d. or s.e.m.

Please complete ALL of the questions below.
Select "Not Applicable" only when the requested information is not relevant for your study.

Materials

Newly Created Materials	Information included in the manuscript?	In which section is the information available? (Reagents and Tools Table, Materials and Methods, Figures, Data Availability Section)
New materials and reagents need to be available; do any restrictions apply?	Not Applicable	

Antibodies	Information included in the manuscript?	In which section is the information available? (Reagents and Tools Table, Materials and Methods, Figures, Data Availability Section)
For **antibodies** provide the following information:<br>- Commercial antibodies: RRID (if possible) or supplier name, catalogue number and or/clone number<br>- Non-commercial: RRID or citation	Yes	Reagents and Tools Table

DNA and RNA sequences	Information included in the manuscript?	In which section is the information available? (Reagents and Tools Table, Materials and Methods, Figures, Data Availability Section)
**Short novel DNA or RNA including primers, probes**: provide the sequences.	Yes	Table EV1

Cell materials	Information included in the manuscript?	In which section is the information available? (Reagents and Tools Table, Materials and Methods, Figures, Data Availability Section)
**Cell lines:** Provide species information, strain. Provide accession number in repository **OR** supplier name, catalog number, clone number, and/**OR** RRID.	Yes	Materials and Methods
**Primary cultures:** Provide species, strain, sex of origin, genetic modification status.	Not Applicable	
Report if the cell lines were recently **authenticated** (e.g., by STR profiling) and tested for mycoplasma contamination.	Yes	Materials and Methods

Experimental animals	Information included in the manuscript?	In which section is the information available? (Reagents and Tools Table, Materials and Methods, Figures, Data Availability Section)
**Laboratory animals or Model organisms:** Provide species, strain, sex, age, genetic modification status. Provide accession number in repository **OR** supplier name, catalog number, clone number, **OR** RRID.	Not Applicable	
**Animal observed in or captured from the field:** Provide species, sex, and age where possible.	Not Applicable	
Please detail **housing and husbandry conditions**.	Not Applicable	

Plants and microbes	Information included in the manuscript?	In which section is the information available? (Reagents and Tools Table, Materials and Methods, Figures, Data Availability Section)
**Plants:** provide species and strain, ecotype and cultivar where relevant, unique accession number if available, and source (including location for collected wild specimens).	Not Applicable	
**Microbes:** provide species and strain, unique accession number if available, and source.	Not Applicable	

Human research participants	Information included in the manuscript?	In which section is the information available? (Reagents and Tools Table, Materials and Methods, Figures, Data Availability Section)
If collected and within the bounds of privacy constraints report on age, sex and gender or ethnicity for all study participants.	Not Applicable	

Core facilities	Information included in the manuscript?	In which section is the information available? (Reagents and Tools Table, Materials and Methods, Figures, Data Availability Section)
If your work benefited from core facilities, was their service mentioned in the acknowledgments section?	Yes	Acknowledgments

Design

Study protocol	Information included in the manuscript?	In which section is the information available? (Reagents and Tools Table, Materials and Methods, Figures, Data Availability Section)
If study protocol has been **pre-registered, provide DOI in the manuscript**. For clinical trials, provide the trial registration number **OR** cite DOI.	Not Applicable	
Report the **clinical trial registration number** (at ClinicalTrials.gov or equivalent), where applicable.	Not Applicable	

Laboratory protocol	Information included in the manuscript?	In which section is the information available? (Reagents and Tools Table, Materials and Methods, Figures, Data Availability Section)
Provide DOI OR other citation details if **external detailed step-by-step protocols** are available.	Yes	Materials and Methods

Experimental study design and statistics	Information included in the manuscript?	In which section is the information available? (Reagents and Tools Table, Materials and Methods, Figures, Data Availability Section)
Include a statement about **sample size** estimate even if no statistical methods were used.	Yes	Figure legends
Were any steps taken to minimize the effects of subjective bias when allocating animals/samples to treatment (e.g. **randomization procedure**)? If yes, have they been described?	Not Applicable	
Include a statement about **blinding** even if no blinding was done.	Yes	Materials and methods
Describe **inclusion/exclusion criteria** if samples or animals were excluded from the analysis. Were the criteria pre-established?<br><br>If sample or data points were omitted from analysis, report if this was due to attrition or intentional exclusion and provide justification.	Not Applicable	
For every figure, are **statistical tests** justified as appropriate? Do the data meet the assumptions of the tests (e.g., normal distribution)? Describe any methods used to assess it. Is there an estimate of variation within each group of data? Is the variance similar between the groups that are being statistically compared?	Yes	Materials and methods

Sample definition and in-laboratory replication	Information included in the manuscript?	In which section is the information available? (Reagents and Tools Table, Materials and Methods, Figures, Data Availability Section)
In the figure legends: state number of times the experiment was **replicated** in laboratory.	Yes	Figure legends
In the figure legends: define whether data describe **technical or biological replicates**.	Yes	Figure legends

Ethics

Ethics	Information included in the manuscript?	In which section is the information available? (Reagents and Tools Table, Materials and Methods, Figures, Data Availability Section)
Studies involving **human participants**: State details of **authority granting ethics approval** (IRB or equivalent committee(s), provide reference number for approval.	Not Applicable	
Studies involving **human participants**: Include a statement confirming that **informed consent** was obtained from all subjects and that the experiments conformed to the principles set out in the WMA Declaration of Helsinki and the Department of Health and Human Services Belmont Report.	Not Applicable	
Studies involving **human participants:** For publication of **patient photos**, include a statement confirming that consent to publish was obtained.	Not Applicable	
Studies involving experimental **animals**: State details of **authority granting ethics approval** (IRB or equivalent committee(s), provide reference number for approval. Include a statement of compliance with ethical regulations.	Not Applicable	
Studies involving **specimen and field samples:** State if relevant **permits** obtained, provide details of authority approving study; if none were required, explain why.	Not Applicable	

Dual Use Research of Concern (DURC)	Information included in the manuscript?	In which section is the information available? (Reagents and Tools Table, Materials and Methods, Figures, Data Availability Section)
Could your study fall under dual use research restrictions? Please check biosecurity documents and list of **select agents and toxins** (CDC): https://www.selectagents.gov/sat/list.htm	Not Applicable	
If you used a select agent, is the security level of the lab appropriate and reported in the manuscript?	Not Applicable	
If a study is subject to dual use research of concern regulations, is the name of the **authority granting approval and reference number** for the regulatory approval provided in the manuscript?	Not Applicable	

Reporting

The MDAR framework recommends adoption of discipline-specific guidelines, established and endorsed through community initiatives. Journals have their own policy about requiring specific guidelines and recommendations to complement MDAR.

Adherence to community standards	Information included in the manuscript?	In which section is the information available? (Reagents and Tools Table, Materials and Methods, Figures, Data Availability Section)
State if relevant guidelines or checklists (e.g., **ICMJE, MIBBI, ARRIVE, PRISMA**) have been followed or provided.	Yes	Materials and Methods
For **tumor marker prognostic studies,** we recommend that you follow the **REMARK** reporting guidelines (see link list at top right). See author guidelines, under 'Reporting Guidelines'. Please confirm you have followed these guidelines.	Not Applicable	
For **phase II and III randomized controlled trials**, please refer to the **CONSORT** flow diagram (see link list at top right) and submit the CONSORT checklist (see link list at top right) with your submission. See author guidelines, under 'Reporting Guidelines'. Please confirm you have submitted this list.	Not Applicable	

Data Availability

Data availability	Information included in the manuscript?	In which section is the information available? (Reagents and Tools Table, Materials and Methods, Figures, Data Availability Section)
Have **primary datasets** been deposited according to the journal's guidelines (see 'Data Deposition' section) and the respective accession numbers provided in the Data Availability Section?	Not Applicable	
Were **human clinical and genomic datasets** deposited in a public access-controlled repository in accordance to ethical obligations to the patients and to the applicable consent agreement?	Not Applicable	
Are **computational models** that are central and integral to a study available without restrictions in a machine-readable form? Were the relevant accession numbers or links provided?	Not Applicable	
If publicly available data were reused, provide the respective **data citations in the reference list**.	Yes	References
